# Characteristics of healthcare personnel with SARS-CoV-2 infection: 10 emerging infections program sites in the United States, April 2020–December 2021

**DOI:** 10.1017/ice.2024.71

**Published:** 2024-05-21

**Authors:** Nora Chea, Taniece Eure, Rebecca Alkis Ramirez, Maria Zlotorzynska, Gregory T. Blazek, Joelle Nadle, Jane Lee, Christopher A. Czaja, Helen Johnston, Devra Barter, Melissa Kellogg, Catherine Emanuel, James Meek, Monica Brackney, Stacy Carswell, Stepy Thomas, Scott K. Fridkin, Lucy E. Wilson, Rebecca Perlmutter, Kaytlynn Marceaux-Galli, Ashley Fell, Sara Lovett, Sarah Lim, Ruth Lynfield, Sarah Shrum Davis, Erin C. Phipps, Marla Sievers, Ghinwa Dumyati, Christopher Myers, Christine Hurley, Erin Licherdell, Rebecca Pierce, Valerie L. S. Ocampo, Eric W. Hall, Christopher Wilson, Cullen Adre, Erika Kirtz, Tiffanie M. Markus, Kathryn Billings, Ian D Plumb, Glen R. Abedi, Jade James-Gist, Shelley S. Magill, Cheri T. Grigg

**Affiliations:** 1 Division of Healthcare Quality Promotion, National Center for Emerging and Zoonotic Infectious Diseases, Centers for Disease Control and Prevention, Atlanta, GA, USA,; 2 Chenega Enterprise Systems and Solutions, LLC, Chesapeake, VA, USA,; 3 California Emerging Infections Program, Oakland, CA, USA,; 4 Colorado Department of Public Health and Environment, Denver, CO, USA,; 5 Connecticut Emerging Infections Program, Yale School of Public Health, New Haven, CT, USA,; 6 Georgia Emerging Infections Program, Atlanta Veterans Affairs Medical Center, Foundation for Atlanta Veterans Education and Research, Atlanta, GA, USA,; 7 Georgia Emerging Infections Program, Emory University School of Medicine, Atlanta, GA, USA,; 8 Maryland Department of Health, Baltimore, MD, USA,; 9 Minnesota Department of Health, St. Paul, MN, USA,; 10 New Mexico Emerging Infections Program, University of New Mexico, Albuquerque, NM, USA,; 11 New Mexico Department of Health, Santa Fe, NM, USA,; 12 New York Emerging Infections Program, University of Rochester Medical Center, Rochester, NY, USA,; 13 Public Health Division, Oregon Health Authority, Portland, OR, USA,; 14 Oregon Health and Science University and Portland State University School of Public Health, Oregon Health and Science University, Portland, OR, USA,; 15 Tennessee Department of Health, Nashville, TN, USA,; 16 Vanderbilt University Medical Center, Nashville, TN, USA; 17 Division of Viral Diseases, National Center for Immunization and Respiratory Diseases, Centers for Disease Control and Prevention, Atlanta, GA, USA

**Keywords:** Healthcare personnel, COVID-19, SARS-CoV-2, social vulnerability

## Abstract

**Background:**

Understanding characteristics of healthcare personnel (HCP) with SARS-CoV-2 infection supports the development and prioritization of interventions to protect this important workforce. We report detailed characteristics of HCP who tested positive for SARS-CoV-2 from April 20, 2020 through December 31, 2021.

**Methods:**

CDC collaborated with Emerging Infections Program sites in 10 states to interview HCP with SARS-CoV-2 infection (case-HCP) about their demographics, underlying medical conditions, healthcare roles, exposures, personal protective equipment (PPE) use, and COVID-19 vaccination status. We grouped case-HCP by healthcare role. To describe residential social vulnerability, we merged geocoded HCP residential addresses with CDC/ATSDR Social Vulnerability Index (SVI) values at the census tract level. We defined highest and lowest SVI quartiles as high and low social vulnerability, respectively.

**Results:**

Our analysis included 7,531 case-HCP. Most case-HCP with roles as certified nursing assistant (CNA) (444, 61.3%), medical assistant (252, 65.3%), or home healthcare worker (HHW) (225, 59.5%) reported their race and ethnicity as either non-Hispanic Black or Hispanic. More than one third of HHWs (166, 45.2%), CNAs (283, 41.7%), and medical assistants (138, 37.9%) reported a residential address in the high social vulnerability category. The proportion of case-HCP who reported using recommended PPE at all times when caring for patients with COVID-19 was lowest among HHWs compared with other roles.

**Conclusions:**

To mitigate SARS-CoV-2 infection risk in healthcare settings, infection prevention, and control interventions should be specific to HCP roles and educational backgrounds. Additional interventions are needed to address high social vulnerability among HHWs, CNAs, and medical assistants.

## Introduction

Healthcare personnel (HCP) played a critical role in combating the COVID-19 pandemic. Protecting HCP from contracting SARS-CoV-2 remains a priority. However, mitigating exposure is a complex challenge. HCP are exposed to SARS-CoV-2 in both workplace and community settings, and several studies have shown that selected groups of HCP have a higher risk of infection than others.^1–10^ In the healthcare workplace, for example, assisting patients with activities of daily living has been shown to be a risk factor for SARS-CoV-2 infection in HCP.^2^ Several studies have suggested community exposures and factors associated with HCP’s living environment may be even more important than workplace exposures.^2,6,11^ We have previously reported that HCP infected with SARS-CoV-2 were more likely to reside in highly socially vulnerable census tracts compared to HCP without SARS-CoV-2, a finding primarily driven by socioeconomic status and household characteristics (e.g., single-parent households, English language proficiency).^11^ Additionally, in a study by Baker et al., Black and multiracial HCP had higher odds of infection with SARS-CoV-2 compared with White HCP.^6^ Better understanding of the occupational and community-related characteristics of HCP who tested positive for SARS-CoV-2 may help inform the development of interventions that account for differences among HCP roles, their community environment, and social vulnerability.

To describe these characteristics of HCP with SARS-CoV-2 infection, we conducted surveillance in 10 Emerging Infections Program (EIP) sites in the United States.^12^ We previously reported the characteristics of 2,625 HCP with SARS-CoV-2 infection between April and November 2020.^13^ Here we provide additional characteristics of HCP with SARS-CoV-2 infection focusing on the demographics, underlying medical conditions, COVID-19 vaccination status, and community and occupational exposures of HCP with SARS-CoV-2 infection in 2020 and 2021 (including the HCP in the previous report), stratified by healthcare roles.

## Methods

### Surveillance setting

The Centers for Disease Control and Prevention collaborated with 10 EIP sites to conduct surveillance of SARS-CoV-2 infections in HCP. Seven of the 10 sites (Colorado, Connecticut, Maryland, Minnesota, New Mexico, Oregon, and Tennessee) recruited a convenience sample of health systems from across the state to participate. Eligible healthcare settings included acute-care hospitals, nursing homes, outpatient clinics, and other outpatient settings (e.g., urgent care clinics, assisted living facilities, home healthcare). New York EIP recruited a convenience sample of health systems in one county to participate, in addition to conducting surveillance of all HCP working in nursing homes in the same county. Two EIP sites (California and Georgia) conducted surveillance of HCP working in any healthcare setting and residing in three California counties in the San Francisco area or in five Georgia counties in the Atlanta area, respectively.

### Definition and ascertainment of case-HCP

HCP were defined as “persons serving in healthcare settings with the potential for direct or indirect exposure to patients or infectious materials including body substances (e.g., blood, tissue, and specific body fluids); contaminated medical supplies, devices, and equipment; contaminated environmental surfaces; or contaminated air”.^14^ Case-HCP were defined as HCP who had a positive SARS-CoV-2 polymerase chain reaction or antigen test result (both of which are hereafter referred to as virus test) from a nasopharyngeal or oral swab from April 20, 2020, through December 31, 2021.

EIP site staff reviewed weekly line lists of HCP with positive virus tests and contacted HCP to conduct a telephone interview in English or Spanish using a standardized questionnaire. EIP staff made at least five contact attempts by telephone, text messages, or email before considering the HCP as non-responsive. To minimize recall bias, EIP site staff aimed to complete interviews within 60 days of the specimen collection date of the positive virus test. A self-administered electronic questionnaire was also available for use at the discretion of EIP sites and participating healthcare systems.

If HCP reported having close contact with patients with COVID-19 in the healthcare setting, interview staff asked questions about personal protective equipment (PPE) use and patient care activities during care of patients with COVID-19. Questions about COVID-19 vaccination status were added to the questionnaire in January 2021. EIP staff verified reported vaccination status and dates of vaccination by reviewing state immunization registries.

Data were collected and managed using REDCap electronic data capture tools hosted at CDC.^15,16^ REDCap (Research Electronic Data Capture) is a secure, web-based software platform designed to support data collection, providing (1) an intuitive interface for validated data capture; (2) audit trails for tracking data manipulation and export procedures; (3) automated export procedures for seamless data downloads to common statistical packages; and (4) procedures for data integration and interoperability with external sources.

### Descriptive and statistical analysis

We grouped case-HCP by the primary role HCP reported working in during the 14 days before collection of the specimen that tested positive for SARS-CoV-2, and by the primary setting where they reported working: hospitals, nursing homes, outpatient clinics, home healthcare, assisted living facilities, or other facilities. Based on the specimen collection dates of the positive virus tests, case-HCP were grouped into 2020 (i.e., before COVID-19 vaccines were available) or 2021 case-HCP (i.e., after COVID-19 vaccines were available). To describe residential social vulnerability of HCP, we merged geocoded residence data for individual HCP with 2020 CDC/ATSDR Social Vulnerability Index (SVI) values at the census tract level. The SVI is “a composite measure used to identify communities most in need of support before, during, and after hazardous events, such as infectious disease outbreaks”.^17,18^ We defined high and low social vulnerability as the highest quartile of SVI (i.e., ≥0.75) and lowest quartile (i.e., ≤0.25), respectively. Analyses were conducted using SAS version 9.4 software (SAS Institute, Cary, NC).

This activity was reviewed by CDC and was conducted in compliance with applicable federal law and CDC policy (45 C.F.R. part 46.102(l)(2), 21 C.F.R. part 56; 42 U.S.C. §241(d); 5 U.S.C. §552a; 44 U.S.C. §3501 et seq.). CDC determined the project was a non-research activity, and no CDC institutional review board (IRB) review was required. IRBs of EIP sites and participating facilities either deemed the project to be a non-research activity not requiring review or provided IRB approval as a research activity.

## Results

A total of 34,179 HCP who tested positive for SARS-CoV-2 were reported to the 10 EIP sites. Of those, 7,637 HCP were enrolled, and 26,542 HCP were not interviewed (details in the Supplementary Appendix). Of the 7,637 HCP enrolled, 106 HCP were excluded from this analysis because their interviews were only partially complete, positive virus test results were from specimens other than nasopharyngeal swab, or the specimen collection dates of the positive nasopharyngeal swabs were missing.

Among 7,531 case-HCP included in this analysis, the median time from specimen collection dates of the positive virus test to interview date was 25 days with an interquartile range of 15 to 43 days. Due to the surge in number of case-HCP during the emergence of the Delta strain of SARS-CoV-2, 433 (5.7%) case-HCP included were interviewed >60 days after the specimen collection date of their positive virus test; we subsequently implemented a rule excluding HCP from interviews if >60 days passed since the specimen collection date of their positive virus test. Additionally, 557 (7.4 %) case-HCP included completed a self-administered questionnaire rather than a telephone interview.

**Figure 1.**
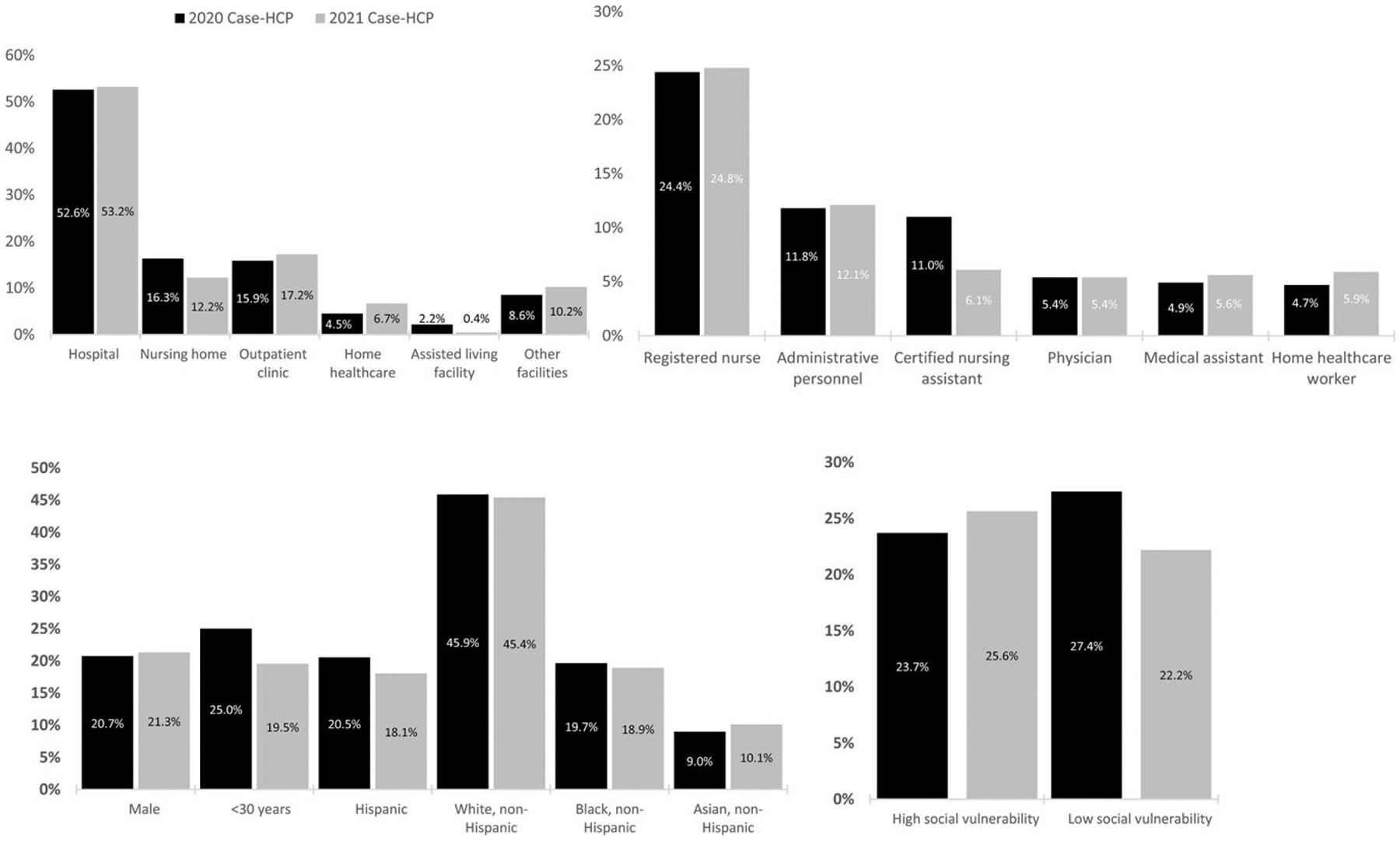
Healthcare personnel with SARS-CoV-2 infection in 2020 and 2021 by setting, role, demographics, and social vulnerability. *Note*: Other facilities include administrative building, correctional facility, dental facility, outpatient dialysis unit, emergency medical service, free-standing emergency room, hospice facility, laboratory, memory care facility, mental health facility, pharmacy, public health department, rehabilitation center, school, COVID-19 testing center, urgent care center.24 HCP did not answer questions about sex or reported sex as unknown.76 HCP did not answer questions about age.168 HCP with missing or unknown ethnicity were grouped as non-Hispanic.592 HCP were not matched with SVI data due to lack of valid addresses or residential addresses that were out of catchment areas.

Overall, 3,975 (52.8%) reported working in hospitals, 1,223 (16.2%) in outpatient clinics, 1,142 (15.2%) in nursing homes, 386 (5.1%) in home healthcare settings, and 126 (1.7%) in assisted living facilities. There were 5,437 case-HCP from 2020 and 2,094 case-HCP from 2021. The distributions of healthcare settings, HCP roles, demographics, and SVI were similar for 2020 and 2021 (Figure 1).

The distribution of case-HCP race and ethnicity, SVI, community exposures, and healthcare settings varied by role (Table 1 and Table S1 in the Supplementary Appendix). Most case-HCP were female (78.8%) and ≥30 years of age (75.5%), and 3,445 case-HCP (45.7%) were non-Hispanic White. Of all case-HCP, 61.9% had at least one underlying condition. Overall, 894 case-HCP (11.9%) reported an administrative role, which included a wide range of roles (e.g., human resources personnel, receptionist, patient service assistant). Among 2,094 case-HCP who tested positive for SARS-CoV-2 in 2021, 1,541 (73.6%) received at least one dose of COVID-19 vaccine ≥14 days before the SARS-CoV-2 positive test dates.

A large proportion of medical assistants (43.8%) and home healthcare workers (HHWs) (38.1%) reported their ethnicity as Hispanic. Black or African American was the most commonly reported race by certified nursing assistants (CNAs) (294, 40.6%). Most medical assistants worked in outpatient clinics (64%), and most CNAs worked in nursing homes (54.6%). More than one third of HHWs (45.2%), CNAs (41.7%), and medical assistants (37.9%) reported a residential address in the highest SVI quartile. In five of ten sites, the HCP role with the highest percentage of personnel living in census tracts in the highest SVI quartile was CNA (Table S2 in the Supplementary Appendix).

Among 2,606 case-HCP who reported having close contact with patients with COVID-19 in healthcare settings, the proportion of HCP who reported using each element of recommended PPE all the time while caring for patients with COVID-19 was lowest among HHWs (i.e., gloves, 72.3%; a mask or respirator, 76.2%; goggles or a face shield, 30.7%; or a gown, 27.7%) compared with registered nurses, administrative personnel, CNAs, physicians, or medical assistants (Tables 2 and S3 in the Supplementary Appendix).

## Discussion

Our analysis included 7,531 HCP who tested positive for SARS-CoV-2 in 10 EIP sites across the United States during 2020 and 2021. These data represent demographics, exposures, PPE use, COVID-19 vaccination status, and residential social vulnerability for a large group of HCP working in a variety of healthcare roles across multiple healthcare settings.

Compared with other healthcare roles, HHWs had the lowest proportion of case-HCP reporting consistent use of each element of recommended PPE^14^ when caring for patients with COVID-19. Medical assistants also reported lower consistent PPE use compared with other healthcare roles. Using all recommended PPE consistently is critical for protecting HCP,^19^ and the lower reported PPE use among case-HCP working in home healthcare, outpatient clinics, and assisted living facilities relative to other settings suggests additional work is needed to determine if these findings were a result of inadequate PPE supplies, access, or training on indications for PPE. Focused assessments may be beneficial to understand how PPE supply needs are determined in these settings, and how PPE is distributed to HCP in different healthcare roles. These findings underscore the need to focus infection prevention and control interventions on HCP in a wide variety of care delivery environments, not limited to hospitals.

Approximately one in three HHWs, medical assistants, or CNAs had not received any doses of COVID-19 vaccines ≥14 days before the SARS-CoV-2 positive test. While COVID-19 vaccines were proven to be very effective in preventing symptomatic COVID-19 among HCP^20^ and HCP were among the priority groups to receive COVID-19 vaccines in early 2021,^21^ COVID-19 vaccine hesitancy was still a challenge among HCP.^21–23^ Due to the potential risks to patients and themselves, focused interventions to reduce COVID-19 vaccine hesitancy should be prioritized for HCP, especially among those who have direct patient contact.

Infection prevention and control staffing, training, and resources are typically less robust in certain healthcare settings, such as home healthcare, outpatient clinics, and assisted living facilities, when compared with hospitals.^24–29^ Project Firstline, an infection control training and education collaborative with public health, academic, and health department partners across the United States, is a CDC-led effort to address this gap.^30^ Providing foundational knowledge of infection prevention and control for all frontline HCP is key, especially recognizing the healthcare workforce includes professionals with a wide range of training and educational backgrounds. Findings from this surveillance activity support the importance of training HCP at greatest risk for SARS-CoV-2 infection and focusing on messaging that is appropriate for specific healthcare settings. It is equally important to engage these HCP, and the organizations that represent them, to better understand the barriers or challenges and potential facilitators of infection prevention and control practices.

Previous work has shown that non-Hispanic Black, non-Hispanic Asian (specifically Filipino), and Hispanic HCP are overrepresented in the long-term care workforce, especially among lower-wage frontline professions.^31,32^ Additionally, racial and ethnic disparities in infection rates have been documented throughout the COVID-19 pandemic.^6,33–35^ In our cohort of HCP with SARS-CoV-2 infection, most HHWs, CNAs, and medical assistants reported their race and ethnicity as either non-Hispanic Black or Hispanic, and an additional 19.6% of HHWs reported their race and ethnicity as non-Hispanic Asian. In our examination of social determinants of health based on the SVI, we found that HHWs, CNAs, and medical assistants were the HCP roles that were most often living in areas with the highest social vulnerability. This is notable, as residential social vulnerability has been identified as an important determinant of risk for SARS-CoV-2 infection.^11,36–39^ A previous case-control analysis using a subset of these data found that HCP with SARS-CoV-2 infection in 2020 were 1.8 times more likely than HCP without SARS-CoV-2 infection to have lived in census tracts with high social vulnerability, with socioeconomic status and household composition driving the disparity.^11^ In that analysis, CNAs and medical assistants were more likely to have lived in high SVI census tracts compared with registered nurses and physicians. This is unsurprising since the mean national salary for these healthcare support occupations is less than $36,000 per year^40^; this is not much more than the 2023 Federal Poverty Level for a family of four ($30,000).^41^

Our findings are subject to three limitations. First, these data were from a convenience sample of healthcare settings and HCP. The results may not be generalizable to all U.S. HCP. Healthcare settings (e.g., hospital, nursing home) were not equally sampled in each site, which may have affected the distribution of HCP roles, and other case-HCP characteristics summarized in our analysis. Second, HCP self-reported their PPE use during care of patients with COVID-19 which could introduce social desirability bias, and further misclassification of consistent use of PPE may have occurred due to the lag time between SARS-CoV-2 virus test specimen collection and the interview. Third, CDC updated the definition of “close contact” with a person with COVID-19 multiple times during the pandemic. To reflect the changes in the definition, the questionnaire was updated three times during the surveillance period. This may have caused misclassification of “close contact” with a COVID-19 patient for some case-HCP, but the impact on the data is expected to be minimal since the main categories of information collected remained the same.

## Conclusion

In conclusion, it is important to recognize and address infection risk among non-physician HCP, and among HCP working in non-hospital settings. In this analysis of 2020–2021 data from one of the largest surveillance systems of U.S. HCP with SARS-CoV-2 infection, HHWs reported the lowest consistent usage of PPE when working with patients with COVID-19. Programs like Project Firstline provide critically needed infection prevention and control training designed to be accessible for all frontline HCP. Most CNAs, medical assistants, and HHWs reported their ethnicity or race as Hispanic or non-Hispanic Black, and more than one third of HCP who reported these three roles also reported living in a census tract with high social vulnerability. More work is needed to understand social and community contributions to infection risk in these vulnerable groups.

## Supplementary Material

Characteristics of HCP with SARS-CoV-2 Supplement

## Figures and Tables

**Table 1.** Characteristics of healthcare personnel with SARS-CoV-2 infection, by primary healthcare role, 2020–2021

	Registered nurse (*n* = 1,846)	Administrative personnel^a^ (*n* = 894)	Certified nursing assistant (*n* = 724)	Physician (*n* = 408)	Medical assistant (*n* = 386)	Home healthcare worker (*n* = 378)	All professions (*N* = 7,531)
Facility type, no. (%)							
Hospital	1,478 (80.1)	389 (43.5)	252 (34.8)	285 (69.9)	78 (20.2)	5 (1.3)	3,975 (52.8)
Nursing home	111 (6.0)	94 (10.5)	395 (54.6)	8 (2.0)	16 (4.1)	72 (19.0)	1,142 (15.2)
Outpatient clinic	153 (8.3)	257 (28.7)	15 (2.1)	98 (24.0)	247 (64.0)	1 (0.3)	1,223 (16.2)
Home healthcare setting	32 (1.7)	12 (1.3)	36 (5.0)	2 (0.5)	0(0.0)	237 (62.7)	386 (5.1)
Assisted living facility	5 (0.3)	13 (1.5)	12 (1.7)	0 (0.0)	7 (1.8)	48 (12.7)	126 (1.7)
Other facilities^b^	67 (3.6)	129 (14.4)	14 (1.9)	15 (3.7)	38 (9.8)	15 (4.0)	679 (9.0)
Sex, no, (%)^c^							
Female	1,587 (86.0)	736 (82.3)	639 (88.3)	200 (49.0)	352 (91.2)	319 (84.4)	5,933 (78.8)
Male	258 (14.0)	156 (17.4)	79 (10.9)	207 (50.7)	32 (8.3)	59 (15.6)	1,574 (20.9)
Age group in years, no. (%)^d^							
Median [IQR]	39 [30–50]	43 [32–53]	38 [27–51]	38 [32–49]	32 [26–41]	46 [33–56]	39 [30–50]
<30	418 (22.6)	158 (17.7)	234 (32.3)	60 (14.7)	155 (40.2)	68 (18.0)	1,769 (23.5)
≥30	1,413 (76.5)	728 (81.4)	482 (66.6)	345 (84.6)	229 (59.3)	304 (80.4)	5,686 (75.5)
Race and ethnicity, no. (%)^e^							
Hispanic	180 (9.8)	224 (25.1)	150 (20.7)	35 (8.6)	169 (43.8)	144 (38.1)	1,495 (19.9)
White, non-Hispanic	1,237 (67.0)	371 (41.5)	148 (20.4)	263 (64.5)	82 (21.2)	31 (8.2)	3,445 (45.7)
Black or African American, non-Hispanic	204 (11.1)	199 (22.3)	294 (40.6)	26 (6.4)	83 (21.5)	81 (21.4)	1,465 (19.5)
Asian, non-Hispanic	149 (8.1)	51 (5.7)	85 (11.7)	67 (16.4)	29 (7.5)	74 (19.6)	700 (9.3)
Other or multiple races	57 (3.1)	34 (3.8)	31 (4.3)	11 (2.7)	12 (3.1)	33 (8.7)	273 (3.6)
Unknown race, non-Hispanic	19 (1.0)	15 (1.7)	16 (2.2)	6 (1.5)	11 (2.8)	15 (4.0)	153 (2.0)
Residential address Social vulnerability index (SVI), no. (%)^f^							
High social vulnerability^g^	225 (13.8)	194 (22.9)	283 (41.7)	29 (7.9)	138 (37.9)	166 (45.2)	1,681 (24.2)
Low social vulnerability^h^	556 (34.1)	175 (20.7)	89 (13.1)	193 (52.6)	55 (15.1)	35 (9.5)	1,804 (26.0)
Community exposures, no. (%)							
Traveled domestically or internationally	356 (19.3)	139 (15.5)	41 (5.7)	104 (25.5)	53 (13.7)	22 (5.8)	1,157 (15.4)
Attended a mass gathering or gathering with people other than household members	512 (27.7)	207 (23.2)	109 (15.1)	136 (33.3)	92 (23.8)	57 (15.1)	1,818 (24.1)
Used public or shared transportation	253 (13.7)	115 (12.9)	84 (11.6)	74 (18.1)	36 (9.3)	71 (18.8)	1,058 (14.1)
Had close contact with ill person(s) outside of a healthcare facility	367 (19.9)	204 (22.8)	109 (15.1)	75 (18.4)	95 (24.6)	50 (13.2)	1,412 (18.8)
Had close contact with a family member(s) who had COVID-19	433 (23.5)	292 (32.7)	117 (16.2)	99 (24.3)	142 (36.8)	88 (23.3)	1,883 (25.0)
Underlying conditions, no. (%)							
At least one underlying condition	1,077 (58.3)	619 (69.2)	500 (69.1)	150 (36.8)	262 (67.9)	263 (69.6)	4,660 (61.9)
Asthma	245 (13.3)	148 (16.6)	110 (15.2)	42 (10.3)	61 (15.8)	50 (13.2)	1,051 (14.0)
Autoimmune or rheumatologic disease	117 (6.3)	37 (4.1)	21 (2.9)	15 (3.7)	18 (4.7)	3 (0.8)	351 (4.7)
Chronic kidney disease	5 (0.3)	3 (0.3)	1 (0.1)	2 (0.5)	4 (1.0)	4 (1.1)	31 (0.4)
Chronic obstructive pulmonary disease	8 (0.4)	6 (0.7)	10 (1.4)	0 (0.0)	0 (0.0)	6 (1.6)	46 (0.6)
Current or recent smoker^i^	375 (20.3)	215 (24.0)	174 (24.0)	26 (6.4)	78 (20.2)	106 (28.0)	1,628 (21.6)
Diabetes mellitus	72 (3.9)	71 (7.9)	63 (8.7)	9 (2.2)	22 (5.7)	45 (11.9)	471 (6.3)
Heart condition	60 (3.3)	37 (4.1)	24 (3.3)	16 (3.9)	10 (2.6)	6 (1.6)	249 (3.3)
Hypertension	260 (14.1)	169 (18.9)	125 (17.3)	46 (11.3)	36 (9.3)	91 (24.1)	1,163 (15.4)
Obesity or severe obesity	515 (27.9)	396 (44.3)	318 (43.9)	43 (10.5)	164 (42.5)	130 (34.4)	2,549 (33.9)
Pregnancy	49 (2.7)	11 (1.2)	14 (1.9)	5 (1.2)	17 (4.4)	7 (1.9)	160 (2.1)
COVID-19 vaccination status at time of test, no. (%)^j^							
Vaccinated	414 (79.6)	176 (69.6)	81 (63.8)	110 (97.3)	72 (61.5)	73 (58.9)	1,541 (73.6)
Unvaccinated	104 (20.0)	77 (30.4)	45 (35.4)	3 (2.7)	45 (38.5)	51 (41.1)	548 (26.2)

a HCP who reported their role as administrative personnel, director, financial personnel, human resources personnel, receptionist, patient service assistant, clinical supervisor, or marketing personnel.

b Other facilities include administrative building, correctional facility, dental facility, outpatient dialysis unit, emergency medical service, free-standing emergency room, hospice facility, laboratory, memory care facility, mental health facility, pharmacy, public health department, rehabilitation center, school, COVID-19 testing center, urgent care center.

c 24 HCP did not answer questions about sex or reported sex as unknown.

d 76 HCP did not answer questions about age.

e 168 HCP with missing or unknown ethnicity were grouped as non-Hispanic.

f Census tract-level SVI for HCP residential address. 592 HCP were not matched with SVI data due to lack of valid addresses, or residential addresses that were outside the catchment areas.

g Highest quartile of SVI values for census tracts where healthcare personnel resided.

h Lowest quartile of SVI values for census tracts where healthcare personnel resided.

i Recent smokers were defined as HCP who quit smoking <1 year before the interview date.

j Among 2,094 case-HCP who tested positive for SARS-CoV-2 in 2021 (registered nurse, *n* = 520; non-clinical administrative worker, *n* = 253; certified nursing assistant, *n* = 127; home healthcare worker, *n* = 124; medical assistant, *n* = 117; physician, *n* = 113; all other professions, *n* = 840); 5 HCP reported unknown COVID-19 vaccination status; vaccinated is defined as having received at least one dose of COVID-19 vaccine ≥14 days before the SARS-CoV-2 positive test dates.

**Table 2.** Personal protective equipment use, workplace exposures, and patient care activities among healthcare personnel with SARS-CoV-2 infection and close contact with patients with COVID-19 in healthcare settings, by primary healthcare role, 2020–2021

	Registered nurse (*N* = 912)	Certified nursing assistant (*N* = 365)	Physician (*N* = 168)	Home healthcare worker (*N* = 101)	Administrative personnel^b^ (*n* = 100)	Medical assistant (*N* = 88)	All professions (*n* = 2,606)
Facility type, no. (%)							
Hospital	787 (86.3)	134 (36.7)	140 (83.3)	1 (1.0)	46 (46.0)	24 (27.3)	1,631 (62.6)
Nursing home	67 (7.3)	210 (57.5)	6 (3.6)	27 (26.7)	19 (19.0)	5 (5.7)	504 (19.3)
Outpatient clinic	18 (2.0)	1 (0.3)	16 (9.5)	0 (0.0)	19 (19.0)	39 (44.3)	156 (6.0)
Home healthcare setting	11 (1.2)	8 (2.2)	0 (0.0)	54 (53.5)	0 (0.0)	0 (0.0)	86 (3.3)
Assisted living facility	2 (0.2)	7 (1.9)	0 (0.0)	16 (15.8)	1 (1.0)	3 (3.4)	42 (1.6)
Other facilities	27 (3.0)	5 (1.4)	6 (3.6)	3 (3.0)	15 (15.0)	17 (19.3)	187 (7.2)
PPE use and exposures in healthcare setting, no. (%)							
Used gloves all the time	840 (92.1)	334 (91.5)	142 (84.5)	73 (72.3)	36 (36.0)	69 (78.4)	2,224 (85.5)
Used a mask or respirator all the time	847 (92.9)	336 (92.1)	156 (92.9)	77 (76.2)	91 (91.0)	83 (94.3)	2,392 (91.8)
Used goggles or a face shield all the time	675 (74.0)	251 (68.8)	126 (75.0)	31 (30.7)	38 (38.0)	55 (62.5)	1,759 (67.7)
Used a gown all the time	638 (70.0)	240 (65.8)	123 (73.2)	28 (27.7)	21 (21.0)	46 (52.3)	1,625 (62.6)
Always cared for COVID-19 patients who had source control in place	149 (16.3)	25 (6.8)	43 (25.6)	8 (7.9)	30 (30.0)	44 (50.0)	527 (20.2)
Had a mucous membrane or skin exposure to body fluids from a COVID-19 patient	164 (18.0)	88 (24.1)	37 (22.0)	34 (33.7)	11 (11.0)	19 (21.6)	495 (19.1)
Practiced extended use or reuse of a respirator	596 (65.4)	192 (52.6)	120 (71.4)	31 (30.7)	30 (30.0)	44 (50.0)	1,507 (57.8)
Always followed hand hygiene recommendations during care of COVID-19 patients	823 (90.2)	339 (92.9)	153 (91.1)	86 (85.1)	86 (86.0)	83 (94.3)	2,346 (90.4)
Patient care activities, no. (%)							
Assisted patients with COVID-19 with activities of daily living	752 (82.5)	344 (94.2)	46 (27.4)	90 (89.1)	13 (13.0)	26 (29.5)	1,716 (65.9)
Bathing^a^	402 (53.5)	268 (77.9)	2 (4.3)	71 (78.9)	0 (0.0)	9 (34.6)	863 (50.3)
Emptying bedpan^a^	413 (54.9)	216 (62.8)	1 (2.2)	25 (27.8)	1 (7.7)	11 (42.3)	766 (44.6)
Feeding^a^	324 (43.1)	260 (75.6)	2 (4.3)	72 (80.0)	1 (7.7)	14 (53.8)	807 (47.0)
Lifting or positioning^a^	715 (95.1)	314 (91.3)	45 (97.8)	64 (71.1)	11 (84.6)	20 (76.9)	1,564 (91.1)
Performing oral care^a^	223 (29.7)	81 (23.5)	1 (2.2)	16 (17.8)	0 (0.0)	6 (23.1)	392 (22.8)
Other activities of daily living^a^	129 (17.2)	82 (23.8)	8 (17.4)	41 (45.6)	6 (46.2)	6 (23.1)	410 (23.9)
Provided non-procedure clinical care to patients with COVID-19	819 (89.8)	287 (78.6)	146 (86.9)	63 (62.4)	24 (24.0)	70 (79.5)	1,963 (75.3)
Performed procedures on patients with COVID-19	724 (79.4)	62 (17.0)	65 (38.7)	5 (5.0)	3 (3.0)	28 (31.8)	1,199 (46.0)
Performed environmental cleaning activities in COVID-19 patient care area	590 (64.7)	307 (84.1)	5 (3.0)	79 (78.2)	5 (5.0)	34 (38.6)	1,277 (49.0)
Provided respiratory care to patients with COVID-19	631 (69.2)	125 (34.2)	70 (41.7)	14 (13.9)	8 (8.0)	28 (31.8)	1,211 (46.5)
Performed administrative activities with patients with COVID-19	23 (2.5)	5 (1.4)	20 (11.9)	3 (3.0)	71 (71.0)	9 (10.2)	217 (8.3)

a Among HCP who assisted patients with COVID-19 with activities of daily living.

b HCP who reported their role as administrative personnel, director, financial personnel, human resources personnel, receptionist, patient service assistant, clinical supervisor, or marketing personnel.
