# Supplementary material for: Characteristics of healthcare personnel with SARS-CoV-2 infection: 10 emerging infections program sites in the United States, April 2020–December 2021
Source: Infect Control Hosp Epidemiol. Author manuscript; Available in PMC 2025 Nov 21. (PMC11518671; doi:10.1017/ice.2024.71)
Supplement: Characteristics of HCP with SARS-CoV-2 Supplement [file NIHMS2009674-supplement-Characteristics_of_HCP_with_SARS-CoV-2_Supplement.pdf]

Supplementary Appendix, Characteristics of Healthcare Personnel with SARS-CoV-2 Infection—10 Emerging Infections Program Sites in the United States, April 2020–December 2021

Supplementary Results

Healthcare personnel who tested positive for SARS-CoV-2 but were not interviewed

Of the 26,542 total healthcare personnel (HCP) with positive SARS-CoV-2 tests who were reported to the Emerging Infections Program (EIP) sites but who were not interviewed: 8,543 HCP (32.2%) could not be reached after at least five contact attempts by telephone, text messages, or email and were considered as non-responsive; 5,497 HCP (20.7%) had dates of specimen collection of the positive virus test >60 days before the interview and were therefore not interviewed to minimize the impact of recall bias; 5,574 HCP (21.0%) were reported during a period when interviews were paused to dedicate health department resources to another priority surveillance activity; 3,572 HCP (13.5%) declined to participate; 335 (1.3%) did not meet the HCP definition; 68 (0.3%) spoke a language other than English or Spanish; 29 HCP (0.1%) resided outside of the surveillance areas; 11 HCP (0.04%) had duplicate reports; four HCP (0.02%) required an interview in Spanish but the interview form had not yet been approved for use; three HCP (0.01%) died before the telephone interview; and for 2,906 HCP (10.9%) the reason was unknown.

Table S1. Characteristics of healthcare personnel with SARS-CoV-2 infection, by primary healthcare role, 2020–2021

	Licensed Practical Nurse (n=253)	Facilities Personnel[k] (n=172)	Nurse Practitioner (n=165)	Environmental Services Personnel[l] (n=163)	Surgical or Medical Technician[m] (n=159)	Food Services Personnel[n] (n=157)	All professions (N=7,531)
**Facility type, no. (%)**							
Hospital	34 (13.4)	121 (70.3)	83 (50.3)	113 (69.3)	107 (67.3)	85 (54.1)	3,975 (52.8)
Nursing home	133 (52.6)	34 (19.8)	16 (9.7)	39 (23.9)	5 (3.1)	61 (38.9)	1,142 (15.2)
Outpatient clinic	42 (16.6)	6 (3.5)	50 (30.3)	6 (3.7)	21 (13.2)	2 (1.3)	1,223 (16.2)
Home healthcare setting	12 (4.7)	2 (1.2)	2 (1.2)	1 (0.6)	0 (0.0)	0 (0.0)	386 (5.1)
Assisted living facility	8 (3.2)	3 (1.7)	0 (0.0)	2 (1.2)	13 (8.2)	6 (3.8)	126 (1.7)
Other facilities[b]	24 (9.5)	6 (3.5)	14 (8.5)	2 (1.2)	13 (8.2)	3 (1.9)	679 (9.0)
**Sex, no, (%)[c]**							
Female	220 (87.0)	64 (37.2)	148 (89.7)	110 (67.5)	119 (74.8)	102 (65.0)	5,933 (78.8)
Male	31 (12.3)	107 (62.2)	16 (9.7)	53 (32.5)	38 (23.9)	55 (35.0)	1,574 (20.9)
**Age group in years, no. (%)[d]**							
Median [IQR]	42 [33–52]	46 [36–56]	41 [34–49]	42 [33–54]	36 [28–45]	41 [28–53]	39 [30–50]
<30	43 (17.0)	22 (12.8)	10 (6.1)	24 (14.7)	46 (28.9)	45 (28.7)	1,769 (23.5)
≥30	205 (81.0)	146 (84.9)	155 (93.9)	136 (83.4)	113 (71.1)	110 (70.1)	5,686 (75.5)
**Race and ethnicity, no. (%)[e]**							
Hispanic	22 (8.7)	59 (34.3)	14 (8.5)	56 (34.4)	42 (26.4)	50 (31.9)	1,495 (19.9)
White, non-Hispanic	92 (36.3)	51 (29.6)	112 (67.9)	36 (22.1)	57 (35.9)	32 (20.4)	3,445 (45.7)
Black or African American, non-Hispanic	91 (36.0)	38 (22.1)	26 (15.8)	50 (30.7)	31 (19.5)	47 (30.0)	1,465 (19.5)
Asian, non-Hispanic	30 (11.9)	14 (8.1)	6 (3.6)	12 (7.4)	17 (10.7)	20 (12.7)	700 (9.3)
Other or multiple races	7 (2.8)	6 (3.6)	7 (4.2)	9 (5.5)	10 (6.2)	6 (3.7)	273 (3.6)
Unknown race, non- Hispanic	11 (4.3)	4 (2.3)	0 (0.0)	0 (0.0)	2 (1.3)	2 (1.3)	153 (2.0)
**Residential address Social Vulnerability Index (SVI), no. (%)[f]**							
High social vulnerability[g]	68 (28.0)	61 (38.4)	5 (3.1)	70 (49.0)	45 (30.8)	63 (45.0)	1,681 (24.2)
Low social vulnerability[h]	40 (16.5)	22 (13.8)	84 (52.8)	13 (9.1)	29 (19.9)	17 (12.1)	1,804 (26.0)
**Community exposures, no. (%)**							
Traveled domestically or internationally	22 (8.7)	14 (8.1)	27 (16.4)	8 (4.9)	21 (13.2)	10 (6.4)	1,157 (15.4)

Attended a mass gathering or gathering with people other than household members	34 (13.4)	27 (15.7)	50 (30.3)	16 (9.8)	38 (23.9)	24 (15.3)	1,818 (24.1)
Used public or shared transportation	20 (7.9)	32 (18.6)	13 (7.9)	30 (18.4)	19 (12.0)	23 (14.7)	1,058 (14.1)
Had close contact with ill person(s) outside of a healthcare facility	29 (11.5)	21 (12.2)	40 (24.2)	25 (15.4)	27 (17.0)	22 (14.0)	1,412 (18.8)
Had close contact with a family member(s) who had COVID-19	40 (15.8)	31 (18.0)	52 (31.5)	35 (21.5)	45 (28.3)	37 (23.6)	1,883 (25.0)
**Underlying conditions, no. (%)**							
At least one underlying condition	183 (72.3)	123 (71.5)	100 (60.6)	111 (68.1)	96 (60.3)	106 (67.5)	4,660 (61.9)
Asthma	48 (19.0)	19 (11.1)	21 (12.7)	18 (11.0)	18 (11.3)	21 (13.4)	1,051 (14.0)
Autoimmune or rheumatologic disease	15 (5.9)	7 (4.1)	15 (9.1)	1 (0.6)	10 (6.3)	1 (0.6)	351 (4.7)
Chronic kidney disease	1 (0.4)	1 (0.6)	1 (0.6)	2 (1.2)	0 (0.0)	0 (0.0)	31 (0.4)
Chronic obstructive pulmonary disease	2 (0.8)	2 (1.2)	0 (0.0)	3 (1.8)	1 (0.6)	1 (0.6)	46 (0.6)
Current or recent smoker[i]	75 (29.6)	59 (34.3)	32 (19.4)	45 (27.6)	35 (22.0)	41 (26.1)	1,628 (21.6)
Diabetes mellitus	31 (12.3)	13 (7.6)	4 (2.4)	19 (11.7)	10 (6.3)	21 (13.4)	471 (6.3)
Heart condition	8 (3.2)	7 (4.1)	9 (5.5)	6 (3.7)	2 (1.3)	6 (3.8)	249 (3.3)
Hypertension	58 (22.9)	42 (24.4)	22 (13.3)	30 (18.4)	24 (15.1)	6 (3.8)	1,163 (15.4)
Obesity or severe obesity	126 (49.8)	70 (40.7)	43 (26.1)	69 (42.3)	59 (37.1)	60 (38.2)	2,549 (33.9)
Pregnancy	3 (1.2)	3 (1.7)	8 (4.9)	1 (0.6)	0 (0.0)	3 (1.9)	160 (2.1)
**COVID-19 vaccination status at time of test, no. (%)[j]**							
Vaccinated	38 (62.3)	26 (66.7)	33 (73.3)	27 (67.5)	30 (83.3)	22 (62.9)	1,541 (73.6)
Unvaccinated	23 (37.7)	13 (33.3)	12 (26.7)	13 (32.5)	6 (16.7)	13 (37.1)	548 (26.2)

Table S1. Characteristics of healthcare personnel with SARS-CoV-2 infection, by primary healthcare role, 2020–2021 (continued)

	Pharmacist[o] (n=144)	Laboratory Personnel[p] (n=141)	Radiology Personnel[q] (n=131)	Physical Therapist (n=126)	Emergency Medical Services Personnel[r] (n=121)	Mental Health Personnel[s] (n=115)	All professions (N=7,531)
**Facility type, no. (%)**							
Hospital	81 (56.3)	101 (71.6)	90 (68.7)	56 (44.4)	39 (32.2)	41 (35.7)	3,975 (52.8)
Nursing home	5 (3.5)	2 (1.4)	0 (0.0)	30 (23.8)	3 (2.5)	4 (3.5)	1,142 (15.2)
Outpatient clinic	16 (11.1)	8 (5.7)	34 (26.0)	28 (22.2)	3 (2.5)	34 (29.6)	1,223 (16.2)
Home healthcare setting	0 (0.0)	0 (0.0)	0 (0.0)	9 (7.1)	0 (0.0)	13 (11.3)	386 (5.1)
Assisted living facility	0 (0.0)	0 (0.0)	0 (0.0)	1 (0.8)	0 (0.0)	0 (0.0)	126 (1.7)
Other facilities[b]	42 (29.1)	30 (21.3)	7 (5.3)	2 (1.6)	76 (62.8)	23 (20.0)	679 (9.0)
**Sex, no, (%)[c]**							
Female	106 (73.6)	98 (69.5)	99 (75.6)	91 (72.2)	42 (34.7)	82 (71.3)	5,933 (78.8)
Male	38 (26.4)	43 (30.5)	32 (24.4)	34 (27.0)	79 (65.3)	32 (27.8)	1,574 (20.9)
**Age group in years, no. (%)[d]**							
Median [IQR]	36 [29–49]	41 [31–52]	40 [31–52]	38 [31–47]	33 [26–43]	35 [28–46]	39 [30–50]
<30	39 (27.1)	32 (22.7)	25 (19.1)	27 (21.4)	46 (38.0)	32 (27.8)	1,769 (23.5)
≥30	104 (72.2)	107 (75.9)	106 (80.9)	98 (77.8)	75 (62.0)	83 (72.2)	5,686 (75.5)
**Race and ethnicity, no. (%)[e]**							
Hispanic	18 (12.5)	26 (18.4)	18 (13.7)	10 (7.9)	26 (21.5)	27 (23.5)	1,495 (19.9)
White, non-Hispanic	80 (55.6)	66 (46.8)	90 (68.7)	95 (75.4)	72 (59.5)	43 (37.4)	3,445 (45.7)
Black or African American, non-Hispanic	21 (14.6)	32 (22.7)	12 (9.1)	6 (4.8)	11 (9.1)	27 (23.5)	1,465 (19.5)
Asian, non-Hispanic	18 (12.5)	12 (8.5)	6 (4.6)	12 (9.5)	6 (5.0)	8 (7.0)	700 (9.3)
Other or multiple races	2 (1.4)	2 (1.4)	2 (1.5)	2 (1.6)	4 (3.3)	5 (4.3)	273 (3.6)
Unknown race, non-Hispanic	5 (3.5)	3 (2.1)	3 (2.3)	1 (0.8)	2 (1.7)	5 (4.3)	153 (2.0)
**Residential address Social Vulnerability Index (SVI), no. (%)[f]**							
High social vulnerability[g]	28 (20.0)	25 (18.5)	21 (18.8)	10 (8.7)	21 (19.1)	23 (20.4)	1,681 (24.2)
Low social vulnerability[h]	41 (29.3)	32 (23.7)	32 (28.6)	45 (39.1)	37 (33.6)	19 (16.8)	1,804 (26.0)
**Community exposures, no. (%)**							
Traveled domestically or internationally	24 (16.7)	29 (20.6)	28 (21.4)	28 (22.2)	14 (11.6)	17 (14.8)	1,157 (15.4)

Attended a mass gathering or gathering with people other than household members	51 (35.4)	40 (28.4)	45 (34.4)	42 (33.3)	32 (26.5)	29 (25.2)	1,818 (24.1)
Used public or shared transportation	23 (16.0)	25 (17.7)	22 (16.8)	22 (17.5)	15 (12.4)	12 (10.4)	1,058 (14.1)
Had close contact with ill person(s) outside of a healthcare facility	36 (25.0)	27 (19.2)	27 (20.6)	15 (11.9)	17 (14.1)	21 (18.3)	1,412 (18.8)
Had close contact with a family member(s) who had COVID-19	45 (31.3)	40 (28.4)	39 (29.8)	21 (16.7)	14 (11.6)	29 (25.2)	1,883 (25.0)
**Underlying conditions, no. (%)**							
At least one underlying condition	77 (53.5)	90 (63.8)	76 (58.0)	57 (45.2)	75 (70.0)	77 (67.0)	4,660 (61.9)
Asthma	20 (13.9)	16 (11.4)	16 (12.2)	11 (8.7)	14 (11.6)	18 (15.7)	1,051 (14.0)
Autoimmune or rheumatologic disease	6 (4.2)	5 (3.6)	12 (9.2)	15 (11.9)	4 (3.3)	8 (7.0)	351 (4.7)
Chronic kidney disease	1 (0.7)	1 (0.7)	1 (0.8)	0 (0.0)	0 (0.0)	2 (1.7)	31 (0.4)
Chronic obstructive pulmonary disease	0 (0.0)	0 (0.0)	1 (0.8)	0 (0.0)	0 (0.0)	1 (0.9)	46 (0.6)
Current or recent smoker[i]	12 (8.3)	28 (19.9)	31 (23.7)	18 (14.3)	31 (25.6)	31 (27.0)	1,628 (21.6)
Diabetes mellitus	9 (6.3)	9 (6.4)	3 (2.3)	3 (2.4)	3 (2.5)	3 (2.6)	471 (6.3)
Heart condition	5 (3.5)	3 (2.1)	4 (3.1)	1 (0.8)	2 (1.7)	4 (3.5)	249 (3.3)
Hypertension	26 (18.1)	27 (19.2)	17 (13.0)	7 (5.6)	9 (7.4)	15 (13.0)	1,163 (15.4)
Obesity or severe obesity	43 (29.9)	46 (32.6)	33 (25.2)	21 (16.7)	41 (33.9)	40 (34.8)	2,549 (33.9)
Pregnancy	6 (4.2)	0 (0.0)	5 (3.8)	6 (4.8)	2 (1.7)	2 (1.7)	160 (2.1)
**COVID-19 vaccination status at time of test, no. (%)[j]**							
Vaccinated	33 (86.8)	42 (77.8)	39 (78.0)	31 (83.8)	35 (72.9)	23 (69.7)	1,541 (73.6)
Unvaccinated	5 (13.2)	12 (22.2)	11 (22.0)	6 (16.2)	12 (25.0)	10 (30.3)	548 (26.2)

Table S1. Characteristics of healthcare personnel with SARS-CoV-2 infection, by primary healthcare role, 2020–2021 (continued)

	Dental practitioner[t] (n=100)	Social worker (n=99)	Respiratory therapist (n=80)	Occupational therapist (n=73)	Phlebotomist (n=67)	All professions (N=7,531)
**Facility type, no. (%)**						
Hospital	3 (3.0)	44 (44.4)	60 (75.0)	29 (39.7)	36 (53.7)	3,975 (52.8)
Nursing home	0 (0.0)	16 (16.2)	12 (15.0)	27 (37.0)	1 (1.5)	1,142 (15.2)
Outpatient clinic	42 (42.0)	20 (20.2)	5 (6.3)	7 (9.6)	22 (32.8)	1,223 (16.2)
Home healthcare setting	0 (0.0)	6 (6.1)	0 (0.0)	5 (6.9)	0 (0.0)	386 (5.1)
Assisted living facility	0 (0.0)	1 (1.0)	0 (0.0)	1 (1.4)	0 (0.0)	126 (1.7)
Other facilities[b]	55 (55.0)	12 (12.1)	3 (3.8)	4 (5.5)	8 (11.9)	679 (9.0)
**Sex, no, (%)[c]**						
Female	89 (89.0)	86 (86.9)	54 (67.5)	64 (87.7)	58 (86.6)	5,933 (78.8)
Male	10 (10.0)	13 (13.1)	26 (32.5)	9 (12.3)	9 (13.4)	1,574 (20.9)
**Age group in years, no. (%)[d]**						
Median [IQR]	33 [26–46]	39 [31–50]	42 [36–51]	37 [30–44]	39 [30–47]	39 [30–50]
<30	41 (41.0)	20 (20.2)	8 (10.0)	17 (23.3)	15 (22.4)	1,769 (23.5)
≥30	57 (57.0)	79 (79.8)	72 (90.0)	56 (76.7)	49 (73.1)	5,686 (75.5)
**Race and ethnicity, no. (%)[e]**						
Hispanic	49 (49.0)	17 (17.2)	10 (12.5)	7 (9.6)	19 (28.4)	1,495 (19.9)
White, non-Hispanic	23 (23.0)	54 (54.6)	46 (57.5)	49 (67.1)	15 (22.4)	3,445 (45.7)
Black or African American, non-Hispanic	9 (9.0)	18 (18.2)	13 (16.3)	11 (15.1)	19 (28.4)	1,465 (19.5)
Asian, non-Hispanic	11 (11.0)	5 (5.1)	6 (7.5)	3 (4.1)	5 (7.5)	700 (9.3)
Other or multiple races	3 (3.0)	3 (3.0)	2 (2.5)	1 (1.4)	7 (10.5)	273 (3.6)
Unknown race, non-Hispanic	5 (5.0)	2 (2.0)	3 (3.8)	2 (2.7)	2 (3.0)	153 (2.0)
**Residential address Social Vulnerability Index (SVI), no. (%)[f]**						
High social vulnerability[g]	24 (25.0)	20 (21.5)	13 (17.8)	11 (16.7)	19 (32.8)	1,681 (24.2)
Low social vulnerability[h]	23 (24.0)	29 (31.2)	23 (31.5)	20 (30.3)	6 (10.3)	1,804 (26.0)
**Community exposures, no. (%)**						
Traveled domestically or internationally	20 (20.0)	19 (19.2)	11 (13.8)	12 (16.4)	14 (20.9)	1,157 (15.4)

Attended a mass gathering or gathering with people other than household members	23 (23.0)	31 (31.3)	18 (22.5)	25 (34.3)	10 (14.9)	1,818 (24.1)
Used public or shared transportation	15 (15.0)	19 (19.2)	7 (8.8)	12 (16.4)	6 (9.0)	1,058 (14.1)
Had close contact with ill person(s) outside of a healthcare facility	16 (16.0)	22 (22.2)	22 (27.5)	12 (16.4)	14 (20.9)	1,412 (18.8)
Had close contact with a family member(s) who had COVID-19	34 (34.0)	30 (30.3)	25 (31.3)	12 (16.4)	21 (31.3)	1,883 (25.0)
**Underlying conditions, no. (%)**						
At least one underlying condition	59 (59.0)	64 (64.7)	54 (67.5)	36 (49.3)	42 (62.7)	4,660 (61.9)
Asthma	12 (12.0)	23 (23.2)	9 (11.3)	8 (11.0)	10 (14.9)	1,051 (14.0)
Autoimmune or rheumatologic disease	2 (2.0)	6 (6.1)	4 (5.0)	4 (5.5)	1 (1.5)	351 (4.7)
Chronic kidney disease	0 (0.0)	1 (1.0)	1 (1.3)	0 (0.0)	0 (0.0)	31 (0.4)
Chronic obstructive pulmonary disease	1 (1.0)	1 (1.0)	1 (1.3)	0 (0.0)	0 (0.0)	46 (0.6)
Current or recent smoker[i]	21 (21.0)	29 (29.3)	20 (25.0)	9 (12.3)	17 (25.4)	1,628 (21.6)
Diabetes mellitus	6 (6.0)	5 (5.1)	5 (6.3)	0 (0.0)	7 (10.5)	471 (6.3)
Heart condition	2 (2.0)	4 (4.0)	4 (5.0)	3 (4.1)	1 (1.5)	249 (3.3)
Hypertension	8 (8.0)	15 (15.2)	15 (18.8)	7 (9.6)	11 (16.4)	1,163 (15.4)
Obesity or severe obesity	31 (31.0)	29 (29.3)	29 (36.3)	20 (27.4)	27 (40.3)	2,549 (33.9)
Pregnancy	3 (3.0)	2 (2.0)	1 (1.3)	2 (2.7)	0 (0.0)	160 (2.1)
**COVID-19 vaccination status at time of test, no. (%)[j]**						
Vaccinated	17 (65.4)	35 (87.5)	11 (64.7)	11 (73.3)	15 (62.5)	1,541 (73.6)
Unvaccinated	9 (34.6)	5 (12.5)	6 (35.3)	4 (26.7)	9 (37.5)	548 (26.2)

Table S1. Characteristics of healthcare personnel with SARS-CoV-2 infection, by primary healthcare role, 2020–2021 (continued)

	Physician assistant (n=64)	Patient care technician or assistant (n=57)	Equipment technician[u] (n=49)	Nurse, other (n=42)	Research personnel (n=39)	All professions (N=7,531)
**Facility type, no. (%)**						
Hospital	40 (62.5)	40 (70.2)	40 (81.6)	27 (64.3)	27 (69.2)	3,975 (52.8)
Nursing home	1 (1.6)	7 (12.3)	0 (0.0)	1 (2.4)	0 (0.0)	1,142 (15.2)
Outpatient clinic	16 (25.0)	3 (5.3)	5 (10.2)	3 (7.1)	9 (23.1)	1,223 (16.2)
Home healthcare setting	1 (1.6)	5 (8.8)	0 (0.0)	3 (7.1)	0 (0.0)	386 (5.1)
Assisted living facility	0 (0.0)	0 (0.0)	0 (0.0)	1 (2.4)	0 (0.0)	126 (1.7)
Other facilities[b]	6 (9.4)	2 (3.5)	4 (8.2)	7 (16.7)	3 (7.7)	679 (9.0)
**Sex, no, (%)[c]**						
Female	45 (70.3)	53 (93.0)	20 (40.8)	38 (90.5)	31 (79.5)	5,933 (78.8)
Male	19 (29.7)	3 (5.3)	29 (59.2)	4 (9.5)	8 (20.5)	1,574 (20.9)
**Age group in years, no. (%)[d]**						
Median [IQR]	36 [30–43]	34 [28–53]	43 [33–50]	41 [37–48]	28 [25–32]	39 [30–50]
<30	14 (21.9)	18 (31.6)	8 (16.3)	1 (2.4)	25 (64.1)	1,769 (23.5)
≥30	49 (76.6)	38 (66.7)	40 (81.6)	41 (97.6)	14 (35.9)	5,686 (75.5)
**Race and ethnicity, no. (%)[e]**						
Hispanic	4 (6.3)	18 (31.6)	13 (26.5)	5 (11.9)	3 (7.7)	1,495 (19.9)
White, non-Hispanic	47 (73.4)	8 (14.0)	19 (38.8)	27 (64.3)	27 (69.2)	3,445 (45.7)
Black or African American, non-Hispanic	5 (7.8)	22 (38.6)	12 (24.5)	7 (16.7)	3 (7.7)	1,465 (19.5)
Asian, non-Hispanic	7 (10.9)	4 (7.0)	2 (4.1)	0 (0.0)	6 (15.4)	700 (9.3)
Other or multiple races	1 (1.6)	3 (5.3)	3 (6.1)	2 (4.8)	0 (0.0)	273 (3.6)
Unknown race, non-Hispanic	0 (0.0)	2 (3.5)	0 (0.0)	1 (2.4)	0 (0.0)	153 (2.0)
**Residential address Social Vulnerability Index (SVI), no. (%)[f]**						
High social vulnerability[g]	3 (4.8)	17 (31.5)	12 (26.7)	5 (12.8)	4 (13.3)	1,681 (24.2)
Low social vulnerability[h]	32 (50.8)	14 (25.9)	8 (17.8)	18 (46.2)	14 (46.7)	1,804 (26.0)
**Community exposures, no. (%)**						

Traveled domestically or internationally	22 (34.4)	7 (12.3)	9 (18.4)	5 (11.9)	14 (35.9)	1,157 (15.4)
Attended a mass gathering or gathering with people other than household members	26 (40.6)	6 (10.6)	14 (28.6)	8 (19.1)	14 (35.9)	1,818 (24.1)
Used public or shared transportation	13 (20.3)	6 (10.5)	9 (18.4)	2 (4.8)	14 (35.9)	1,058 (14.1)
Had close contact with ill person(s) outside of a healthcare facility	13 (20.3)	9 (15.8)	10 (20.4)	10 (23.8)	10 (25.6)	1,412 (18.8)
Had close contact with a family member(s) who had COVID-19	16 (25.0)	12 (21.1)	13 (26.5)	12 (28.6)	18 (46.2)	1,883 (25.0)
**Underlying conditions, no. (%)**						
At least one underlying condition	30 (46.9)	41 (71.9)	33 (67.4)	27 (64.3)	19 (48.7)	4,660 (61.9)
Asthma	13 (20.3)	11 (19.3)	3 (6.1)	6 (14.3)	4 (10.3)	1,051 (14.0)
Autoimmune or rheumatologic disease	5 (7.8)	1 (1.8)	1 (2.0)	2 (4.8)	1 (2.6)	351 (4.7)
Chronic kidney disease	0 (0.0)	0 (0.0)	0 (0.0)	0 (0.0)	0 (0.0)	31 (0.4)
Chronic obstructive pulmonary disease	0 (0.0)	0 (0.0)	0 (0.0)	0 (0.0)	0 (0.0)	46 (0.6)
Current or recent smoker[i]	4 (6.3)	14 (24.6)	13 (26.5)	5 (11.9)	7 (18.0)	1,628 (21.6)
Diabetes mellitus	0 (0.0)	6 (10.5)	4 (8.2)	1 (2.4)	0 (0.0)	471 (6.3)
Heart condition	3 (4.7)	2 (3.5)	2 (4.1)	1 (2.4)	5 (12.8)	249 (3.3)
Hypertension	1 (1.6)	10 (17.5)	5 (10.2)	4 (9.5)	2 (5.1)	1,163 (15.4)
Obesity or severe obesity	7 (10.9)	30 (52.6)	18 (36.7)	19 (45.2)	11 (28.2)	2,549 (33.9)
Pregnancy	5 (7.8)	0 (0.0)	0 (0.0)	0 (0.0)	1 (2.6)	160 (2.1)
**COVID-19 vaccination status at time of test, no. (%)[j]**						
Vaccinated	20 (87.0)	14 (70.0)	10 (71.4)	8 (100)	10 (100)	1,541 (73.6)
Unvaccinated	3 (13.0)	6 (30.0)	4 (28.6)	0 (0.0)	0 (0.0)	548 (26.2)

Table S1. Characteristics of healthcare personnel with SARS-CoV-2 infection, by primary healthcare role, 2020–2021 (continued)

	Student (n=36)	Transport personnel (n=36)	Dietician[v] (n=34)	Other licensed healthcare professional (n=31)	Coordinator or case manager (n=29)	All professions (N=7,531)
**Facility type, no. (%)**						
Hospital	24 (66.7)	26 (72.2)	21 (61.8)	10 (32.3)	11 (37.9)	3,975 (52.8)
Nursing home	2 (5.6)	1 (2.8)	8 (23.5)	1 (3.2)	2 (6.9)	1,142 (15.2)
Outpatient clinic	6 (16.7)	3 (8.3)	3 (8.8)	18 (58.1)	5 (17.2)	1,223 (16.2)
Home healthcare setting	0 (0.0)	0 (0.0)	0 (0.0)	0 (0.0)	3 (10.3)	386 (5.1)
Assisted living facility	0 (0.0)	1 (2.8)	0 (0.0)	0 (0.0)	3 (10.3)	126 (1.7)
Other facilities[b]	4 (11.1)	5 (13.9)	2 (5.9)	2 (6.5)	5 (17.2)	679 (9.0)
**Sex, no, (%)[c]**						
Female	31 (86.1)	17 (47.2)	34 (100)	22 (71.0)	25 (86.2)	5,933 (78.8)
Male	5 (13.9)	19 (52.8)	0 (0.0)	9 (29.0)	4 (13.8)	1,574 (20.9)
**Age group in years, no. (%)[d]**						
Median [IQR]	25 [24–29]	41 [26–55]	38 [28–49]	39 [31–54]	42 [35–50]	39 [30–50]
<30	27 (75.0)	14 (38.9)	12 (35.3)	5 (16.1)	2 (6.9)	1,769 (23.5)
≥30	9 (25.0)	22 (61.1)	22 (64.7)	26 (83.9)	27 (93.1)	5,686 (75.5)
**Race and ethnicity, no. (%)[e]**						
Hispanic	5 (13.9)	6 (16.7)	5 (14.7)	4 (12.9)	11 (37.9)	1,495 (19.9)
White, non-Hispanic	20 (55.6)	9 (25.0)	22 (64.7)	21 (67.7)	10 (34.5)	3,445 (45.7)
Black or African American, non-Hispanic	5 (13.9)	15 (41.7)	3 (8.8)	2 (6.5)	5 (17.2)	1,465 (19.5)
Asian, non-Hispanic	4 (11.1)	3 (8.3)	2 (5.9)	3 (9.7)	2 (6.9)	700 (9.3)
Other or multiple races	0 (0.0)	0 (0.0)	0 (0.0)	1 (3.2)	1 (3.5)	273 (3.6)
Unknown race, non-Hispanic	2 (5.6)	3 (8.3)	2 (5.9)	0 (0.0)	0 (0.0)	153 (2.0)
**Residential address Social Vulnerability Index (SVI), no. (%)[f]**						
High social vulnerability[g]	3 (8.6)	12 (37.5)	6 (18.8)	2 (7.1)	8 (28.6)	1,681 (24.2)
Low social vulnerability[h]	13 (37.1)	7 (21.9)	10 (31.3)	8 (28.6)	6 (21.4)	1,804 (26.0)
**Community exposures, no. (%)**						
Traveled domestically or internationally	6 (16.7)	0 (0.0)	5 (14.7)	6 (19.4)	5 (17.2)	1,157 (15.4)

Attended a mass gathering or gathering with people other than household members	10 (27.8)	5 (13.9)	13 (38.2)	8 (25.8)	7 (24.1)	1,818 (24.1)
Used public or shared transportation	4 (11.1)	3 (8.3)	5 (14.7)	3 (9.7)	5 (17.2)	1,058 (14.1)
Had close contact with ill person(s) outside of a healthcare facility	4 (11.1)	4 (11.1)	7 (20.6)	5 (16.1)	7 (24.1)	1,412 (18.8)
Had close contact with a family member(s) who had COVID-19	8 (22.2)	8 (22.2)	12 (35.3)	7 (22.6)	12 (41.4)	1,883 (25.0)
**Underlying conditions, no. (%)**						
At least one underlying condition	10 (27.8)	21 (58.3)	19 (55.9)	15 (48.4)	22 (75.9)	4,660 (61.9)
Asthma	3 (8.3)	2 (5.6)	7 (20.6)	4 (12.9)	5 (17.2)	1,051 (14.0)
Autoimmune or rheumatologic disease	0 (0.0)	1 (2.8)	4 (11.8)	0 (0.0)	2 (6.9)	351 (4.7)
Chronic kidney disease	0 (0.0)	0 (0.0)	0 (0.0)	0 (0.0)	0 (0.0)	31 (0.4)
Chronic obstructive pulmonary disease	0 (0.0)	1 (2.8)	0 (0.0)	0 (0.0)	1 (3.5)	46 (0.6)
Current or recent smoker[i]	1 (2.8)	5 (13.9)	5 (14.7)	6 (19.4)	9 (31.0)	1,628 (21.6)
Diabetes mellitus	0 (0.0)	5 (13.9)	4 (11.8)	0 (0.0)	3 (10.3)	471 (6.3)
Heart condition	1 (2.8)	2 (5.6)	1 (2.9)	0 (0.0)	2 (6.9)	249 (3.3)
Hypertension	1 (2.8)	7 (19.4)	4 (11.8)	2 (6.5)	5 (17.2)	1,163 (15.4)
Obesity or severe obesity	6 (16.7)	17 (47.2)	5 (14.7)	6 (19.4)	13 (44.8)	2,549 (33.9)
Pregnancy	0 (0.0)	0 (0.0)	3 (8.8)	1 (3.2)	0 (0.0)	160 (2.1)
**COVID-19 vaccination status at time of test, no. (%)[j]**						
Vaccinated	5 (62.5)	4 (44.4)	7 (87.5)	6 (42.9)	6 (66.7)	1,541 (73.6)
Unvaccinated	3 (37.5)	5 (55.6)	1 (12.5)	8 (57.1)	3 (33.3)	548 (26.2)

Table S1. Characteristics of healthcare personnel with SARS-CoV-2 infection, by primary healthcare role, 2020–2021 (continue)

	Speech therapist (n=27)	Patient screener or greeter (n=22)	Activities coordinator (n=15)	Chaplain (n=10)	Other professions or preferred not to answer (n=138)	All professions (N=7,531)
**Facility type, no. (%)**						
Hospital	12 (44.4)	15 (68.2)	0 (0.0)	7 (70.0)	65 (47.1)	3,975 (52.8)
Nursing home	8 (29.6)	0 (0.0)	12 (80.0)	1 (10.0)	14 (10.1)	1,142 (15.2)
Outpatient clinic	6 (22.2)	5 (22.7)	0 (0.0)	0 (0.0)	24 (17.4)	1,223 (16.2)
Home healthcare setting	0 (0.0)	0 (0.0)	0 (0.0)	0 (0.0)	5 (3.6)	386 (5.1)
Assisted living facility	0 (0.0)	0 (0.0)	1 (6.7)	0 (0.0)	0 (0.0)	126 (1.7)
Other facilities[b]	1 (3.7)	2 (9.1)	2 (13.3)	2 (20.0)	30 (21.7)	679 (9.0)
**Sex, no, (%)[c]**						
Female	25 (92.6)	14 (63.6)	12 (80.0)	5 (50.0)	96 (69.6)	5,933 (78.8)
Male	2 (7.4)	8 (36.4)	3 (20.0)	5 (50.0)	40 (29.0)	1,574 (20.9)
**Age group in years, no. (%)[d]**						
Median [IQR]	37 [32–48]	30 [22–44]	33 [28–58]	58 [38–69]	36 [29–50]	39 [30–50]
<30	5 (18.5)	11 (50.0)	6 (40.0)	1 (10.0)	35 (25.4)	1,769 (23.5)
≥30	22 (81.5)	11 (50.0)	9 (60.0)	9 (90.0)	95 (68.8)	5,686 (75.5)
**Race and ethnicity, no. (%)[e]**						
Hispanic	3 (11.1)	8 (36.4)	5 (33.3)	0 (0.0)	33 (23.9)	1,495 (19.9)
White, non-Hispanic	18 (66.7)	8 (36.4)	4 (26.7)	9 (90.0)	51 (37.0)	3,445 (45.7)
Black or African American, non-Hispanic	3 (11.1)	3 (13.6)	2 (13.3)	1 (10.0)	28 (20.3)	1,465 (19.5)
Asian, non-Hispanic	3 (11.1)	2 (9.1)	3 (20.0)	0 (0.0)	13 (9.4)	700 (9.3)
Other or multiple races	0 (0.0)	1 (4.6)	1 (6.7)	0 (0.0)	4 (2.9)	273 (3.6)
Unknown race, non-Hispanic	0 (0.0)	0 (0.0)	0 (0.0)	0 (0.0)	9 (6.5)	153 (2.0)
**Residential address Social Vulnerability Index (SVI), no. (%)[f]**						
High social vulnerability[g]	2 (8.3)	7 (36.8)	7 (50.0)	2 (22.2)	29 (22.3)	1,681 (24.2)
Low social vulnerability[h]	11 (45.8)	3 (15.8)	1 (7.1)	3 (33.3)	41 (31.5)	1,804 (26.0)
**Community exposures, no. (%)**						
Traveled domestically or internationally	7 (25.9)	5 (22.7)	3 (20.0)	4 (40.0)	26 (18.8)	1,157 (15.4)
Attended a mass gathering or gathering	8 (29.6)	2 (9.1)	5 (33.3)	4 (40.0)	40 (29.2)	1,818 (24.1)

with people other than household members						
Used public or shared transportation	7 (25.9)	4 (18.2)	2 (13.3)	4 (40.0)	29 (21.0)	1,058 (14.1)
Had close contact with ill person(s) outside of a healthcare facility	8 (29.6)	5 (22.7)	2 (13.3)	1 (10.0)	24 (17.5)	1,412 (18.8)
Had close contact with a family member(s) who had COVID-19	6 (22.2)	5 (22.7)	1 (6.7)	1 (10.0)	31 (22.5)	1,883 (25.0)
**Underlying conditions, no. (%)**						
At least one underlying condition	13 (48.2)	10 (45.5)	14 (93.3)	7 (70.0)	82 (59.4)	4,660 (61.9)
Asthma	3 (11.1)	0 (0.0)	4 (26.7)	2 (20.0)	26 (18.8)	1,051 (14.0)
Autoimmune or rheumatologic disease	2 (7.4)	1 (4.6)	0 (0.0)	0 (0.0)	4 (2.9)	351 (4.7)
Chronic kidney disease	0 (0.0)	0 (0.0)	0 (0.0)	0 (0.0)	0 (0.0)	31 (0.4)
Chronic obstructive pulmonary disease	0 (0.0)	0 (0.0)	0 (0.0)	0 (0.0)	0(0.0)	46 (0.6)
Current or recent smoker[i]	2 (7.4)	5 (22.7)	7 (46.7)	3 (30.0)	34 (24.6)	1,628 (21.6)
Diabetes mellitus	0 (0.0)	1 (4.6)	2 (13.3)	0 (0.0)	10 (7.3)	471 (6.3)
Heart condition	1 (3.7)	0 (0.0)	0 (0.0)	2 (20.0)	3 (2.2)	249 (3.3)
Hypertension	4 (14.8)	1 (4.6)	4 (26.7)	3 (30.0)	19 (13.8)	1,163 (15.4)
Obesity or severe obesity	6 (22.2)	6 (27.3)	9 (60.0)	1 (10.0)	42 (30.4)	2,549 (33.9)
Pregnancy	0 (0.0)	0 (0.0)	0 (0.0)	0 (0.0)	0 (0.0)	160 (2.1)
**COVID-19 vaccination status at time of test, no. (%)[j]**						
Vaccinated	6 (85.7)	6 (75.0)	5 (83.3)	4 (80.0)	36 (67.9)	1,541 (73.6)
Unvaccinated	1 (14.3)	2 (25.0)	1 (16.7)	1 (20.0)	16 (30.2)	548 (26.2)

[a]HCP who reported their role as administrative personnel, director, financial personnel, human resources personnel, receptionist, patient service assistant, clinical supervisor, or marketing personnel.

[b]Other facilities include: administrative building, correctional facility, dental facility, outpatient dialysis unit, emergency medical service, free standing emergency room, hospice facility, laboratory, memory care facility, mental health facility, pharmacy, public health department, rehabilitation center, school, COVID-19 testing center, urgent care center.

[c]24 HCP did not answer question about sex or reported sex as unknown.

[d]76 HCP did not answer question about age.

[e]168 HCP with missing or unknown ethnicity were grouped as non-Hispanic.

[f]Census tract-level SVI for HCP residential address. 592 HCP were not matched with SVI data due to lack of valid addresses or residential addresses that were out of catchment areas.

[g]Highest quartile of SVI values for census tracts where healthcare personnel resided.

[h]Lowest quartile of SVI values for census tracts where healthcare personnel resided.
[i]Recent smokers were defined as HCP who quit smoking <1 year before the interview date.
[j]Among 2,094 HCP cases who tested positive for SARS-CoV-2 in 2021; 5 HCP reported unknown COVID-19 vaccination status; vaccinated is defined as having received at least one dose of COVID-19 vaccine ≥14 days before the SARS-CoV-2 positive test dates.
[k]HCP who reported their role as facilities personnel, electrician, or engineer.
[l]HCP who reported their role as environmental services, laundry, or housekeeping personnel.
[m]HCP who reported their role as surgical or medical technician, ambulatory technician, dialysis technician, or radiologist therapist.
[n]HCP who reported their role as food services personnel, chef, or dietary aide.
[o]HCP who reported their role as pharmacist or pharmacist interns.
[p]HCP who reported their role as laboratory personnel, histotechnologist, or laboratory supervisor.
[q]HCP who reported their role as radiology personnel, cardiac sonographer, CT technician, MRI technician, mammographer, or X-ray technician.
[r]HCP who reported their role as emergency medical services personnel, paramedic personnel, fire fighter, national guard, or emergency medical technician.
[s]HCP who reported their role as mental health personnel, behavioral technician, addictions counselor, mental health technician, or clinical psychologist.
[t]HCP who reported their role as dental practitioner, dental assistant, dental hygienist, or hygienist assistant.
[u]HCP who reported their role as equipment technician, biomedical equipment technician, cardiac monitor technician, supply technician, telemetry technician, or sterile processing technician.
[v]HCP who reported their role as dietitian or nutrition assistant.

Table S2. Percentage of healthcare personnel with SARS-CoV-2 infection living in high or low Social Vulnerability Index census tracts, by Emerging Infections Program site and primary healthcare role, 2020–2021

Site, no. (%)		All Professions[a]	Registered Nurse	Administrative Personnel[b]	Certified Nursing Assistant	Physician	Medical Assistant	Home Healthcare worker	Other Professions[c]
EIP[d] 1	High social vulnerability[e]	50 (16.2)	10 (9.9)	6 (18.2)	6 (30.0)	2 (6.7)	3 (18.8)	1 (20.0)	22 (21.2)
	Low social vulnerability[f]	113 (36.6)	45 (44.6)	9 (27.3)	6 (30.0)	17 (56.7)	4 (25.0)	1 (20.0)	31 (29.8)
EIP 2	High social vulnerability	60 (21.0)	15 (15.6)	7 (14.9)	5 (41.7)	0 (0.0)	7 (50.0)	0 (0.0)	26 (24.1)
	Low social vulnerability	49 (17.1)	22 (22.9)	7 (14.9)	1 (8.3)	4 (50.0)	2 (14.3)	0 (0.0)	13 (12.0)
EIP 3	High social vulnerability	59 (18.0)	15 (13.2)	7 (21.9)	4 (44.4)	5 (12.8)	6 (40.0)	1 (100)	21 (17.8)
	Low social vulnerability	130 (39.6)	53 (46.5)	8 (25.0)	4 (44.4)	14 (35.9)	4 (26.7)	0 (0.0)	47 (39.8)
EIP 4	High social vulnerability	231 (16.9)	38 (13.1)	40 (18.9)	33 (27.0)	2 (3.6)	21 (25.0)	8 (25.0)	89 (15.6)
	Low social vulnerability	472 (34.6)	123 (42.6)	57 (26.9)	23 (18.9)	35 (62.5)	18 (21.4)	8 (25.0)	208 (36.5)
EIP 5	High social vulnerability	130 (23.2)	23 (13.5)	15 (24.2)	8 (21.6)	2 (9.5)	28 (50.9)	--	54 (25.1)
	Low social vulnerability	163 (29.1)	62 (36.5)	10 (16.1)	7 (18.9)	11 (52.4)	11 (20.0)	--	62 (28.8)
EIP 6	High social vulnerability	26 (12.1)	3 (4.2)	6 (17.6)	5 (33.3)	3 (25.0)	0 (0.0)	--	9 (11.5)
	Low social vulnerability	97 (45.1)	40 (56.3)	13 (38.2)	7 (46.7)	6 (50.0)	2 (40.0)	--	29 (37.2)
EIP 7	High social vulnerability	105 (16.0)	26 (14.4)	12 (13.8)	12 (27.9)	2 (3.3)	2 (25.0)	0 (0.0)	51 (18.5)
	Low social vulnerability	247 (37.6)	72 (39.8)	24 (27.6)	6 (14.0)	42 (68.9)	1 (12.5)	0 (0.0)	102 (37.0)
EIP 8	High social vulnerability	240 (27.1)	13 (6.9)	20 (25.3)	116 (58.3)	3 (7.0)	3 (23.1)	1 (100)	84 (23.3)
	Low social vulnerability	216 (24.4)	63 (33.3)	20 (25.3)	21 (10.6)	19 (44.2)	3 (23.1)	0 (0.0)	90 (24.9)
EIP 9	High social vulnerability	689 (35.1)	62 (22.3)	73 (32.7)	81 (41.3)	7 (9.5)	67 (45.9)	155 (47.5)	244 (34.0)
	Low social vulnerability	263 (13.4)	51 (18.3)	23 (10.3)	13 (6.6)	37 (50.0)	8 (5.5)	26 (8.0)	105 (14.6)
EIP 10	High social vulnerability	91 (24.4)	20 (13.9)	8 (21.6)	13 (50.0)	3 (13.0)	1 (12.5)	--	46 (34.1)
	Low social vulnerability	54 (14.5)	25 (17.4)	4 (10.8)	1 (3.8)	8 (34.8)	2 (25.0)	--	14 (10.4)
All EIP sites	High social vulnerability	1,681 (24.2)	225 (13.8)	194 (22.9)	283 (41.7)	29 (7.9)	138 (37.9)	166 (45.2)	646 (24.1)
	Low social vulnerability	1,804 (26.0)	556 (34.1)	175 (20.7)	89 (13.1)	193 (52.6)	55 (15.1)	35 (9.5)	701 (26.1)

[a]592 HCP were not matched with SVI data due to lack of valid addresses or residential addresses that were out of catchment areas.
[b]HCP who reported their role as administrative personnel, director, financial personnel, human resources personnel, receptionist, patient service assistant, clinical supervisor, or marketing personnel.
[c]All other professions include: roles such as licensed practical nurse, nurse practitioner, facilities personnel, environmental services personnel, food services personnel, surgical or medical technician, laboratory personnel, pharmacist, or radiology personnel.
[d]Emerging Infections Program.

[e]Highest quartile of SVI values for census tracts where healthcare personnel resided.
[f]Lowest quartile of SVI values for census tracts where healthcare personnel resided.

Table S3. Personal protective equipment use, workplace exposures, and patient care activities among healthcare personnel with SARS-CoV-2 infection and close contact with patients with COVID-19 in healthcare settings, by primary healthcare role, 2020–2021

	Licensed Practical Nurse (n=121)	Nurse Practitioner (n=73)	Surgical or Medical Technicians[b] (n=72)	Environmental Services Personnel[c] (n=28)	Facilities Personnel[d] (n=27)	Food Services Personnel[e] (n=7)	All professions (n=2,606)
**Facility type, no. (%)**							
Hospital	19 (15.7)	37 (50.7)	60 (83.3)	16 (57.1)	23 (85.2)	3 (42.9)	1,631 (62.6)
Nursing home	73 (60.3)	8 (11.0)	2 (2.8)	11 (39.3)	2 (7.4)	2 (28.6)	504 (19.3)
Outpatient clinic	7 (5.8)	15 (20.6)	3 (4.2)	1 (3.6)	2 (7.4)	0 (0.0)	156 (6.0)
Home healthcare setting	2 (1.7)	1 (1.4)	0 (0.0)	0 (0.0)	0 (0.0)	0 (0.0)	86 (3.3)
Assisted living facility	4 (3.3)	0 (0.0)	4 (5.6)	0 (0.0)	0 (0.0)	2 (28.6)	42 (1.6)
Other facilities	16 (13.2)	12 (16.4)	3 (4.2)	0 (0.0)	0 (0.0)	0 (0.0)	187 (7.2)
**PPE use and exposures in healthcare setting, no. (%)**							
Used gloves all the time	107 (88.4)	63 (86.3)	67 (93.1)	26 (92.9)	22 (81.5)	5 (71.4)	2,224 (85.5)
Used a mask or respirator all the time	109 (90.1)	63 (86.3)	68 (94.4)	27 (96.4)	26 (96.3)	0 (0.0)	2,392 (91.8)
Used goggles or a face shield all the time	82 (67.8)	56 (76.7)	57 (79.2)	17 (60.7)	17 (63.0)	3 (42.9)	1,759 (67.7)
Used a gown all the time	79 (65.3)	52 (71.2)	50 (70.4)	19 (67.9)	14 (51.9)	5 (71.4)	1,625 (62.6)
Always cared for COVID-19 patients who had source control in place	16 (13.2)	27 (37.0)	31 (43.1)	2 (7.1)	8 (29.6)	1 (14.3)	527 (20.2)
Had a mucous membrane or skin exposure to body fluids from a COVID-19 patient	21 (17.4)	12 (16.9)	9 (12.5)	1 (3.6)	1 (3.7)	1 (14.3)	495 (19.1)
Practiced extended use or reuse of a respirator	58 (47.9)	49 (67.1)	46 (63.9)	12 (42.9)	7 (25.9)	0 (0.0)	1,507 (57.8)
Always followed hand hygiene recommendations during care of COVID-19	108 (89.3)	65 (91.6)	66 (91.7)	25 (89.3)	23 (85.2)	6 (85.7)	2,346 (90.4)

patients							
**Patient care activities, no. (%)**							
Assisted patients with COVID-19 with activities of daily living	85 (70.3)	18 (24.7)	42 (58.3)	4 (14.3)	8 (29.6)	3 (42.9)	1,716 (65.9)
Bathing[a]	41 (48.2)	5 (27.8)	12 (28.6)	0 (0.0)	0 (0.0)	0 (0.0)	863 (50.3)
Emptying bedpan[a]	34 (40.0)	2 (11.1)	14 (33.3)	3 (75.0)	2 (25.0)	0 (0.0)	766 (44.6)
Feeding[a]	49 (57.7)	6 (33.3)	10 (23.8)	1 (25.0)	0 (0.0)	0 (0.0)	807 (47.0)
Lifting or positioning[a]	79 (92.9)	0 (0.0)	39 (92.9)	0 (0.0)	2 (25.0)	0 (0.0)	1,564 (91.1)
Performing oral care[a]	18 (21.2)	2 (11.1)	5 (11.9)	0 (0.0)	0 (0.0)	0 (0.0)	392 (22.8)
Other activities of daily living[a]	12 (14.1)	3 (16.7)	17 (40.5)	0 (0.0)	5 (62.5)	1 (33.3)	410 (23.9)
Provided non-procedure clinical care to patients with COVID-19	111 (91.7)	64 (87.7)	42 (58.3)	2 (7.1)	4 (14.8)	0 (0.0)	1,963 (75.3)
Performed procedures on patients with COVID-19	76 (62.8)	19 (26.0)	44 (61.1)	0 (0.0)	0 (0.0)	0 (0.0)	1,199 (46.0)
Performed environmental cleaning activities in COVID-19 patient care area	64 (52.9)	8 (11.0)	28 (38.9)	26 (92.9)	12 (44.4)	0 (0.0)	1,277 (49.0)
Provided respiratory care to patients with COVID-19	72 (59.5)	34 (46.6)	26 (36.1)	0 (0.0)	0 (0.0)	0 (0.0)	1,211 (46.5)
Performed administrative activities with patients with COVID-19	3 (2.5)	4 (5.5)	2 (2.8)	0 (0.0)	8 (29.6)	5 (71.4)	217 (8.3)

[a]Among HCP who assisted patients with COVID-19 with activities of daily living.

[b]HCP who reported their role as surgical or medical technician, ambulatory technician, dialysis technician, or radiologist therapist.

[c]HCP who reported their role as environmental services, laundry, or housekeeping personnel.

[d]HCP who reported their role as facilities personnel, electrician, or engineer.

[e]HCP who reported their role as food services personnel, chef, or dietary aide.
