# Supplementary material for: Characteristics of healthcare personnel with SARS-CoV-2 infection: 10 emerging infections program sites in the United States, April 2020–December 2021
Source: Infect Control Hosp Epidemiol. 2024 May 21;45(9):1090–8. doi: 10.1017/ice.2024.71 (PMC11518671; doi:10.1017/ice.2024.71)
Supplement: Supplementary file 1 [file S0899823X24000710sup001.docx]

A total of 8,543 HCP could not be reached by telephone; 5,497 HCP had dates of specimen collection of the positive virus test >60 days before the interview; 5,574 HCP were reported during a period when interviews were paused to dedicate health department resources to another priority surveillance activity; 3,572 HCP declined to participate; 335 individuals did not meet the HCP definition; 68 spoke a language other than English or Spanish; 29 HCP resided outside of the surveillance areas;  11 HCP had duplicate reports; four HCP required an interview in Spanish but the interview form had not yet been approved for use; three HCP died before the telephone interview; and for 2,906 HCP the reason was unknown.
