# Supplementary material for: Characteristics of healthcare personnel with SARS-CoV-2 infection: 10 emerging infections program sites in the United States, April 2020–December 2021
Source: Infect Control Hosp Epidemiol. 2024 May 21;45(9):1090–8. doi: 10.1017/ice.2024.71 (PMC11518671; doi:10.1017/ice.2024.71)
Supplement: Supplementary file 2 [file S0899823X24000710sup002.docx]

**Supplementary Results**

**Healthcare personnel** **who tested positive for SARS-CoV-2** **but were not interviewed**

Of the 26,542 total healthcare personnel (HCP) with positive SARS-CoV-2 tests who were reported to the Emerging Infections Program (EIP) sites but who were not interviewed: 8,543 HCP (32.2%) could not be reached after at least five contact attempts by telephone, text messages, or email and were considered as non-responsive; 5,497 HCP (20.7%) had dates of specimen collection of the positive virus test >60 days before the interview and were therefore not interviewed to minimize the impact of recall bias; 5,574 HCP (21.0%) were reported during a period when interviews were paused to dedicate health department resources to another priority surveillance activity; 3,572 HCP (13.5%) declined to participate; 335 (1.3%) did not meet the HCP definition; 68 (0.3%) spoke a language other than English or Spanish; 29 HCP (0.1%) resided outside of the surveillance areas;  11 HCP (0.04%) had duplicate reports; four HCP (0.02%) required an interview in Spanish but the interview form had not yet been approved for use; three HCP (0.01%) died before the telephone interview; and for 2,906 HCP (10.9%) the reason was unknown. 

Table S1. Characteristics of healthcare personnel with SARS-CoV-2 infection, by primary healthcare role, 2020–2021

|  | **Licensed Practical Nurse** **(n=****253)** | **Facilities Personnel**^**k**^ **(n=172)** | **Nurse Practitioner** **(n=165)** | **Environmental Services Personnel**^**l**^ **(n=****163)** | **Surgical or Medical Technician**^**m**^ **(n=****159)** | **Food Services Personnel**^**n**^ **(n=****157)** | **All professions** **(N=7,531)** |  |
| --- | --- | --- | --- | --- | --- | --- | --- | --- |
| **Facility type, no. (%)** |  |  |  |  |  |  |  |  |
| Hospital | 34 (13.4) | 121 (70.3) | 83 (50.3) | 113 (69.3) | 107 (67.3) | 85 (54.1) | 3,975 (52.8) |  |
| Nursing home | 133 (52.6) | 34 (19.8) | 16 (9.7) | 39 (23.9) | 5 (3.1) | 61 (38.9) | 1,142 (15.2) |  |
| Outpatient clinic | 42 (16.6) | 6 (3.5) | 50 (30.3) | 6 (3.7) | 21 (13.2) | 2 (1.3) | 1,223 (16.2) |  |
| Home healthcare setting | 12 (4.7) | 2 (1.2) | 2 (1.2) | 1 (0.6) | 0 (0.0) | 0 (0.0) | 386 (5.1) |  |
| Assisted living facility | 8 (3.2) | 3 (1.7) | 0 (0.0) | 2 (1.2) | 13 (8.2) | 6 (3.8) | 126 (1.7) |  |
| Other facilities^b^ | 24 (9.5) | 6 (3.5) | 14 (8.5) | 2 (1.2) | 13 (8.2) | 3 (1.9) | 679 (9.0) |  |
| **Sex, no, (%)**^**c**^ |  |  |  |  |  |  |  |  |
| Female | 220 (87.0) | 64 (37.2) | 148 (89.7) | 110 (67.5) | 119 (74.8) | 102 (65.0) | 5,933 (78.8) |  |
| Male | 31 (12.3) | 107 (62.2) | 16 (9.7) | 53 (32.5) | 38 (23.9) | 55 (35.0) | 1,574 (20.9) |  |
| **Age group in years, no. (%)**^**d**^ |  |  |  |  |  |  |  |  |
| Median [IQR] | 42 [33–52] | 46 [36–56] | 41 [34–49] | 42 [33–54] | 36 [28–45] | 41 [28–53] | 39 [30–50] |  |
| <30 | 43 (17.0) | 22 (12.8) | 10 (6.1) | 24 (14.7) | 46 (28.9) | 45 (28.7) | 1,769 (23.5) |  |
| ≥30 | 205 (81.0) | 146 (84.9) | 155 (93.9) | 136 (83.4) | 113 (71.1) | 110 (70.1) | 5,686 (75.5) |  |
| **Race and ethnicity, no. (%)**^**e**^ |  |  |  |  |  |  |  |  |
| Hispanic | 22 (8.7) | 59 (34.3) | 14 (8.5) | 56 (34.4) | 42 (26.4) | 50 (31.9) | 1,495 (19.9) |  |
| White, non-Hispanic | 92 (36.3) | 51 (29.6) | 112 (67.9) | 36 (22.1) | 57 (35.9) | 32 (20.4) | 3,445 (45.7) |  |
| Black or African American,        non-Hispanic | 91 (36.0) | 38 (22.1) | 26 (15.8) | 50 (30.7) | 31 (19.5) | 47 (30.0) | 1,465 (19.5) |  |
| Asian, non-Hispanic | 30 (11.9) | 14 (8.1) | 6 (3.6) | 12 (7.4) | 17 (10.7) | 20 (12.7) | 700 (9.3) |  |
| Other or multiple races | 7 (2.8) | 6 (3.6) | 7 (4.2) | 9 (5.5) | 10 (6.2) | 6 (3.7) | 273 (3.6) |  |
| Unknown race, non- Hispanic | 11 (4.3) | 4 (2.3) | 0 (0.0) | 0 (0.0) | 2 (1.3) | 2 (1.3) | 153 (2.0) |  |
| **Residential address Social Vulnerability Index (SVI), no. (%)**^**f**^ |  |  |  |  |  |  |  |  |
| High social vulnerability^g^ | 68 (28.0) | 61 (38.4) | 5 (3.1) | 70 (49.0) | 45 (30.8) | 63 (45.0) | 1,681 (24.2) |  |
| Low social vulnerability^h^ | 40 (16.5) | 22 (13.8) | 84 (52.8) | 13 (9.1) | 29 (19.9) | 17 (12.1) | 1,804 (26.0) |  |
| **Community exposures, no. (%)** |  |  |  |  |  |  |  |  |
| Traveled domestically or      internationally | 22 (8.7) | 14 (8.1) | 27 (16.4) | 8 (4.9) | 21 (13.2) | 10 (6.4) | 1,157 (15.4) |  |
| Attended a mass gathering or      gathering with people other      than household members | 34 (13.4) | 27 (15.7) | 50 (30.3) | 16 (9.8) | 38 (23.9) | 24 (15.3) | 1,818 (24.1) |  |
| Used public or shared      transportation | 20 (7.9) | 32 (18.6) | 13 (7.9) | 30 (18.4) | 19 (12.0) | 23 (14.7) | 1,058 (14.1) |  |
| Had close contact with ill      person(s) outside of a      healthcare facility | 29 (11.5) | 21 (12.2) | 40 (24.2) | 25 (15.4) | 27 (17.0) | 22 (14.0) | 1,412 (18.8) |  |
| Had close contact with a      family member(s) who      had COVID-19 | 40 (15.8) | 31 (18.0) | 52 (31.5) | 35 (21.5) | 45 (28.3) | 37 (23.6) | 1,883 (25.0) |  |
| **Underlying conditions, no. (%)** |  |  |  |  |  |  |  |  |
| At least one underlying      condition | 183 (72.3) | 123 (71.5) | 100 (60.6) | 111 (68.1) | 96 (60.3) | 106 (67.5) | 4,660 (61.9) |  |
| Asthma | 48 (19.0) | 19 (11.1) | 21 (12.7) | 18 (11.0) | 18 (11.3) | 21 (13.4) | 1,051 (14.0) |  |
| Autoimmune or      rheumatologic disease | 15 (5.9) | 7 (4.1) | 15 (9.1) | 1 (0.6) | 10 (6.3) | 1 (0.6) | 351 (4.7) |  |
| Chronic kidney disease | 1 (0.4) | 1 (0.6) | 1 (0.6) | 2 (1.2) | 0 (0.0) | 0 (0.0) | 31 (0.4) |  |
| Chronic obstructive      pulmonary disease | 2 (0.8) | 2 (1.2) | 0 (0.0) | 3 (1.8) | 1 (0.6) | 1 (0.6) | 46 (0.6) |  |
| Current or recent smoker^i^ | 75 (29.6) | 59 (34.3) | 32 (19.4) | 45 (27.6) | 35 (22.0) | 41 (26.1) | 1,628 (21.6) |  |
| Diabetes mellitus | 31 (12.3) | 13 (7.6) | 4 (2.4) | 19 (11.7) | 10 (6.3) | 21 (13.4) | 471 (6.3) |  |
| Heart condition | 8 (3.2) | 7 (4.1) | 9 (5.5) | 6 (3.7) | 2 (1.3) | 6 (3.8) | 249 (3.3) |  |
| Hypertension | 58 (22.9) | 42 (24.4) | 22 (13.3) | 30 (18.4) | 24 (15.1) | 6 (3.8) | 1,163 (15.4) |  |
| Obesity or severe obesity | 126 (49.8) | 70 (40.7) | 43 (26.1) | 69 (42.3) | 59 (37.1) | 60 (38.2) | 2,549 (33.9) |  |
| Pregnancy | 3 (1.2) | 3 (1.7) | 8 (4.9) | 1 (0.6) | 0 (0.0) | 3 (1.9) | 160 (2.1) |  |
| **COVID-19 vaccination status at time of test, no. (%)**^**j**^ |  |  |  |  |  |  |  |  |
| Vaccinated | 38 (62.3) | 26 (66.7) | 33 (73.3) | 27 (67.5) | 30 (83.3) | 22 (62.9) | 1,541 (73.6) |  |
| Unvaccinated | 23 (37.7) | 13 (33.3) | 12 (26.7) | 13 (32.5) | 6 (16.7) | 13 (37.1) | 548 (26.2) |  |

Table S1. Characteristics of healthcare personnel with SARS-CoV-2 infection, by primary healthcare role, 2020–2021 (continued)

|  | **Pharmacist**^**o**^ **(n=144)** | **Laboratory Personnel**^**p**^ **(n=****141)** | **Radiology Personnel**^**q**^ **(n=131)** | **Physical Therapist**  **(n=****126)** | **Emergency Medical Services Personnel**^**r**^ **(n=****121)** | **Mental Health Personnel**^**s**^ **(n=****115)** | **All professions** **(N=7,531)** |
| --- | --- | --- | --- | --- | --- | --- | --- |
| **Facility type, no. (%)** |  |  |  |  |  |  |  |
| Hospital | 81 (56.3) | 101 (71.6) | 90 (68.7) | 56 (44.4) | 39 (32.2) | 41 (35.7) | 3,975 (52.8) |
| Nursing home | 5 (3.5) | 2 (1.4) | 0 (0.0) | 30 (23.8) | 3 (2.5) | 4 (3.5) | 1,142 (15.2) |
| Outpatient clinic | 16 (11.1) | 8 (5.7) | 34 (26.0) | 28 (22.2) | 3 (2.5) | 34 (29.6) | 1,223 (16.2) |
| Home healthcare setting | 0 (0.0) | 0 (0.0) | 0 (0.0) | 9 (7.1) | 0 (0.0) | 13 (11.3) | 386 (5.1) |
| Assisted living facility | 0 (0.0) | 0 (0.0) | 0 (0.0) | 1 (0.8) | 0 (0.0) | 0 (0.0) | 126 (1.7) |
| Other facilities^b^ | 42 (29.1) | 30 (21.3) | 7 (5.3) | 2 (1.6) | 76 (62.8) | 23 (20.0) | 679 (9.0) |
| **Sex, no, (%)**^**c**^ |  |  |  |  |  |  |  |
| Female | 106 (73.6) | 98 (69.5) | 99 (75.6) | 91 (72.2) | 42 (34.7) | 82 (71.3) | 5,933 (78.8) |
| Male | 38 (26.4) | 43 (30.5) | 32 (24.4) | 34 (27.0) | 79 (65.3) | 32 (27.8) | 1,574 (20.9) |
| **Age group in years, no. (%)**^**d**^ |  |  |  |  |  |  |  |
| Median [IQR] | 36 [29–49] | 41 [31–52] | 40 [31–52] | 38 [31–47] | 33 [26–43] | 35 [28–46] | 39 [30–50] |
| <30 | 39 (27.1) | 32 (22.7) | 25 (19.1) | 27 (21.4) | 46 (38.0) | 32 (27.8) | 1,769 (23.5) |
| ≥30 | 104 (72.2) | 107 (75.9) | 106 (80.9) | 98 (77.8) | 75 (62.0) | 83 (72.2) | 5,686 (75.5) |
| **Race and ethnicity, no. (%)**^**e**^ |  |  |  |  |  |  |  |
| Hispanic | 18 (12.5) | 26 (18.4) | 18 (13.7) | 10 (7.9) | 26 (21.5) | 27 (23.5) | 1,495 (19.9) |
| White, non-Hispanic | 80 (55.6) | 66 (46.8) | 90 (68.7) | 95 (75.4) | 72 (59.5) | 43 (37.4) | 3,445 (45.7) |
| Black or African American,        non-Hispanic | 21 (14.6) | 32 (22.7) | 12 (9.1) | 6 (4.8) | 11 (9.1) | 27 (23.5) | 1,465 (19.5) |
| Asian, non-Hispanic | 18 (12.5) | 12 (8.5) | 6 (4.6) | 12 (9.5) | 6 (5.0) | 8 (7.0) | 700 (9.3) |
| Other or multiple races | 2 (1.4) | 2 (1.4) | 2 (1.5) | 2 (1.6) | 4 (3.3) | 5 (4.3) | 273 (3.6) |
| Unknown race, non-Hispanic | 5 (3.5) | 3 (2.1) | 3 (2.3) | 1 (0.8) | 2 (1.7) | 5 (4.3) | 153 (2.0) |
| **Residential address Social Vulnerability Index (SVI), no. (%)**^**f**^ |  |  |  |  |  |  |  |
| High social vulnerability^g^ | 28 (20.0) | 25 (18.5) | 21 (18.8) | 10 (8.7) | 21 (19.1) | 23 (20.4) | 1,681 (24.2) |
| Low social vulnerability^h^ | 41 (29.3) | 32 (23.7) | 32 (28.6) | 45 (39.1) | 37 (33.6) | 19 (16.8) | 1,804 (26.0) |
| **Community exposures, no. (%)** |  |  |  |  |  |  |  |
| Traveled domestically or      internationally | 24 (16.7) | 29 (20.6) | 28 (21.4) | 28 (22.2) | 14 (11.6) | 17 (14.8) | 1,157 (15.4) |
| Attended a mass gathering or      gathering with people other      than household members | 51 (35.4) | 40 (28.4) | 45 (34.4) | 42 (33.3) | 32 (26.5) | 29 (25.2) | 1,818 (24.1) |
| Used public or shared      transportation | 23 (16.0) | 25 (17.7) | 22 (16.8) | 22 (17.5) | 15 (12.4) | 12 (10.4) | 1,058 (14.1) |
| Had close contact with ill      person(s) outside of a healthcare      facility | 36 (25.0) | 27 (19.2) | 27 (20.6) | 15 (11.9) | 17 (14.1) | 21 (18.3) | 1,412 (18.8) |
| Had close contact with a family      member(s) who had COVID-19 | 45 (31.3) | 40 (28.4) | 39 (29.8) | 21 (16.7) | 14 (11.6) | 29 (25.2) | 1,883 (25.0) |
| **Underlying conditions, no. (%)** |  |  |  |  |  |  |  |
| At least one underlying      condition | 77 (53.5) | 90 (63.8) | 76 (58.0) | 57 (45.2) | 75 (70.0) | 77 (67.0) | 4,660 (61.9) |
| Asthma | 20 (13.9) | 16 (11.4) | 16 (12.2) | 11 (8.7) | 14 (11.6) | 18 (15.7) | 1,051 (14.0) |
| Autoimmune or rheumatologic      disease | 6 (4.2) | 5 (3.6) | 12 (9.2) | 15 (11.9) | 4 (3.3) | 8 (7.0) | 351 (4.7) |
| Chronic kidney disease | 1 (0.7) | 1 (0.7) | 1 (0.8) | 0 (0.0) | 0 (0.0) | 2 (1.7) | 31 (0.4) |
| Chronic obstructive pulmonary      disease | 0 (0.0) | 0 (0.0) | 1 (0.8) | 0 (0.0) | 0 (0.0) | 1 (0.9) | 46 (0.6) |
| Current or recent smoker^i^ | 12 (8.3) | 28 (19.9) | 31 (23.7) | 18 (14.3) | 31 (25.6) | 31 (27.0) | 1,628 (21.6) |
| Diabetes mellitus | 9 (6.3) | 9 (6.4) | 3 (2.3) | 3 (2.4) | 3 (2.5) | 3 (2.6) | 471 (6.3) |
| Heart condition | 5 (3.5) | 3 (2.1) | 4 (3.1) | 1 (0.8) | 2 (1.7) | 4 (3.5) | 249 (3.3) |
| Hypertension | 26 (18.1) | 27 (19.2) | 17 (13.0) | 7 (5.6) | 9 (7.4) | 15 (13.0) | 1,163 (15.4) |
| Obesity or severe obesity | 43 (29.9) | 46 (32.6) | 33 (25.2) | 21 (16.7) | 41 (33.9) | 40 (34.8) | 2,549 (33.9) |
| Pregnancy | 6 (4.2) | 0 (0.0) | 5 (3.8) | 6 (4.8) | 2 (1.7) | 2 (1.7) | 160 (2.1) |
| **COVID-19 vaccination status at time of test, no. (%)**^**j**^ |  |  |  |  |  |  |  |
| Vaccinated | 33 (86.8) | 42 (77.8) | 39 (78.0) | 31 (83.8) | 35 (72.9) | 23 (69.7) | 1,541 (73.6) |
| Unvaccinated | 5 (13.2) | 12 (22.2) | 11 (22.0) | 6 (16.2) | 12 (25.0) | 10 (30.3) | 548 (26.2) |

Table S1. Characteristics of healthcare personnel with SARS-CoV-2 infection, by primary healthcare role, 2020–2021 (continued)

|  | **Dental practitioner**^**t**^ **(n=100)** | **Social worker (n=99)** | **Respiratory therapist** **(n=80)** | **Occupational therapist** **(n=73)** | **Phlebotomist** **(n=67)** | **All professions** **(N=7,531)** |
| --- | --- | --- | --- | --- | --- | --- |
| **Facility type, no. (%)** |  |  |  |  |  |  |
| Hospital | 3 (3.0) | 44 (44.4) | 60 (75.0) | 29 (39.7) | 36 (53.7) | 3,975 (52.8) |
| Nursing home | 0 (0.0) | 16 (16.2) | 12 (15.0) | 27 (37.0) | 1 (1.5) | 1,142 (15.2) |
| Outpatient clinic | 42 (42.0) | 20 (20.2) | 5 (6.3) | 7 (9.6) | 22 (32.8) | 1,223 (16.2) |
| Home healthcare setting | 0 (0.0) | 6 (6.1) | 0 (0.0) | 5 (6.9) | 0 (0.0) | 386 (5.1) |
| Assisted living facility | 0 (0.0) | 1 (1.0) | 0 (0.0) | 1 (1.4) | 0 (0.0) | 126 (1.7) |
| Other facilities^b^ | 55 (55.0) | 12 (12.1) | 3 (3.8) | 4 (5.5) | 8 (11.9) | 679 (9.0) |
| **Sex, no, (%)**^**c**^ |  |  |  |  |  |  |
| Female | 89 (89.0) | 86 (86.9) | 54 (67.5) | 64 (87.7) | 58 (86.6) | 5,933 (78.8) |
| Male | 10 (10.0) | 13 (13.1) | 26 (32.5) | 9 (12.3) | 9 (13.4) | 1,574 (20.9) |
| **Age group in years, no. (%)**^**d**^ |  |  |  |  |  |  |
| Median [IQR] | 33 [26–46] | 39 [31–50] | 42 [36–51] | 37 [30–44] | 39 [30–47] | 39 [30–50] |
| <30 | 41 (41.0) | 20 (20.2) | 8 (10.0) | 17 (23.3) | 15 (22.4) | 1,769 (23.5) |
| ≥30 | 57 (57.0) | 79 (79.8) | 72 (90.0) | 56 (76.7) | 49 (73.1) | 5,686 (75.5) |
| **Race and ethnicity, no. (%)**^**e**^ |  |  |  |  |  |  |
| Hispanic | 49 (49.0) | 17 (17.2) | 10 (12.5) | 7 (9.6) | 19 (28.4) | 1,495 (19.9) |
| White, non-Hispanic | 23 (23.0) | 54 (54.6) | 46 (57.5) | 49 (67.1) | 15 (22.4) | 3,445 (45.7) |
| Black or African American, non-      Hispanic | 9 (9.0) | 18 (18.2) | 13 (16.3) | 11 (15.1) | 19 (28.4) | 1,465 (19.5) |
| Asian, non-Hispanic | 11 (11.0) | 5 (5.1) | 6 (7.5) | 3 (4.1) | 5 (7.5) | 700 (9.3) |
| Other or multiple races | 3 (3.0) | 3 (3.0) | 2 (2.5) | 1 (1.4) | 7 (10.5) | 273 (3.6) |
| Unknown race, non-Hispanic | 5 (5.0) | 2 (2.0) | 3 (3.8) | 2 (2.7) | 2 (3.0) | 153 (2.0) |
| **Residential address Social Vulnerability Index (SVI), no. (%)**^**f**^ |  |  |  |  |  |  |
| High social vulnerability^g^ | 24 (25.0) | 20 (21.5) | 13 (17.8) | 11 (16.7) | 19 (32.8) | 1,681 (24.2) |
| Low social vulnerability^h^ | 23 (24.0) | 29 (31.2) | 23 (31.5) | 20 (30.3) | 6 (10.3) | 1,804 (26.0) |
| **Community exposures, no. (%)** |  |  |  |  |  |  |
| Traveled domestically or      internationally | 20 (20.0) | 19 (19.2) | 11 (13.8) | 12 (16.4) | 14 (20.9) | 1,157 (15.4) |
| Attended a mass gathering or      gathering with people other than        household members | 23 (23.0) | 31 (31.3) | 18 (22.5) | 25 (34.3) | 10 (14.9) | 1,818 (24.1) |
| Used public or shared transportation | 15 (15.0) | 19 (19.2) | 7 (8.8) | 12 (16.4) | 6 (9.0) | 1,058 (14.1) |
| Had close contact with ill person(s)      outside of a healthcare facility | 16 (16.0) | 22 (22.2) | 22 (27.5) | 12 (16.4) | 14 (20.9) | 1,412 (18.8) |
| Had close contact with a family      member(s) who had COVID-19 | 34 (34.0) | 30 (30.3) | 25 (31.3) | 12 (16.4) | 21 (31.3) | 1,883 (25.0) |
| **Underlying conditions, no. (%)** |  |  |  |  |  |  |
| At least one underlying      condition | 59 (59.0) | 64 (64.7) | 54 (67.5) | 36 (49.3) | 42 (62.7) | 4,660 (61.9) |
| Asthma | 12 (12.0) | 23 (23.2) | 9 (11.3) | 8 (11.0) | 10 (14.9) | 1,051 (14.0) |
| Autoimmune or rheumatologic disease | 2 (2.0) | 6 (6.1) | 4 (5.0) | 4 (5.5) | 1 (1.5) | 351 (4.7) |
| Chronic kidney disease | 0 (0.0) | 1 (1.0) | 1 (1.3) | 0 (0.0) | 0 (0.0) | 31 (0.4) |
| Chronic obstructive pulmonary disease | 1 (1.0) | 1 (1.0) | 1 (1.3) | 0 (0.0) | 0 (0.0) | 46 (0.6) |
| Current or recent smoker^i^ | 21 (21.0) | 29 (29.3) | 20 (25.0) | 9 (12.3) | 17 (25.4) | 1,628 (21.6) |
| Diabetes mellitus | 6 (6.0) | 5 (5.1) | 5 (6.3) | 0 (0.0) | 7 (10.5) | 471 (6.3) |
| Heart condition | 2 (2.0) | 4 (4.0) | 4 (5.0) | 3 (4.1) | 1 (1.5) | 249 (3.3) |
| Hypertension | 8 (8.0) | 15 (15.2) | 15 (18.8) | 7 (9.6) | 11 (16.4) | 1,163 (15.4) |
| Obesity or severe obesity | 31 (31.0) | 29 (29.3) | 29 (36.3) | 20 (27.4) | 27 (40.3) | 2,549 (33.9) |
| Pregnancy | 3 (3.0) | 2 (2.0) | 1 (1.3) | 2 (2.7) | 0 (0.0) | 160 (2.1) |
| **COVID-19 vaccination status at time of test, no. (%)**^**j**^ |  |  |  |  |  |  |
| Vaccinated | 17 (65.4) | 35 (87.5) | 11 (64.7) | 11 (73.3) | 15 (62.5) | 1,541 (73.6) |
| Unvaccinated | 9 (34.6) | 5 (12.5) | 6 (35.3) | 4 (26.7) | 9 (37.5) | 548 (26.2) |

Table S1. Characteristics of healthcare personnel with SARS-CoV-2 infection, by primary healthcare role, 2020–2021 (continued) 

|  | **Physician assistant (n=64)** | **Patient care technician or assistant** **(n=57)** | **Equipment technician**^**u**^ **(n=49)** | **Nurse, other** **(n=42)** | **Research personnel (n=39)** | **All professions** **(N=7,531)** |
| --- | --- | --- | --- | --- | --- | --- |
| **Facility type, no. (%)** |  |  |  |  |  |  |
| Hospital | 40 (62.5) | 40 (70.2) | 40 (81.6) | 27 (64.3) | 27 (69.2) | 3,975 (52.8) |
| Nursing home | 1 (1.6) | 7 (12.3) | 0 (0.0) | 1 (2.4) | 0 (0.0) | 1,142 (15.2) |
| Outpatient clinic | 16 (25.0) | 3 (5.3) | 5 (10.2) | 3 (7.1) | 9 (23.1) | 1,223 (16.2) |
| Home healthcare setting | 1 (1.6) | 5 (8.8) | 0 (0.0) | 3 (7.1) | 0 (0.0) | 386 (5.1) |
| Assisted living facility | 0 (0.0) | 0 (0.0) | 0 (0.0) | 1 (2.4) | 0 (0.0) | 126 (1.7) |
| Other facilities^b^ | 6 (9.4) | 2 (3.5) | 4 (8.2) | 7 (16.7) | 3 (7.7) | 679 (9.0) |
| **Sex, no, (%)**^**c**^ |  |  |  |  |  |  |
| Female | 45 (70.3) | 53 (93.0) | 20 (40.8) | 38 (90.5) | 31 (79.5) | 5,933 (78.8) |
| Male | 19 (29.7) | 3 (5.3) | 29 (59.2) | 4 (9.5) | 8 (20.5) | 1,574 (20.9) |
| **Age group in years, no. (%)**^**d**^ |  |  |  |  |  |  |
| Median [IQR] | 36 [30–43] | 34 [28–53] | 43 [33–50] | 41 [37–48] | 28 [25–32] | 39 [30–50] |
| <30 | 14 (21.9) | 18 (31.6) | 8 (16.3) | 1 (2.4) | 25 (64.1) | 1,769 (23.5) |
| ≥30 | 49 (76.6) | 38 (66.7) | 40 (81.6) | 41 (97.6) | 14 (35.9) | 5,686 (75.5) |
| **Race and ethnicity, no. (%)**^**e**^ |  |  |  |  |  |  |
| Hispanic | 4 (6.3) | 18 (31.6) | 13 (26.5) | 5 (11.9) | 3 (7.7) | 1,495 (19.9) |
| White, non-Hispanic | 47 (73.4) | 8 (14.0) | 19 (38.8) | 27 (64.3) | 27 (69.2) | 3,445 (45.7) |
| Black or African American,        non-Hispanic | 5 (7.8) | 22 (38.6) | 12 (24.5) | 7 (16.7) | 3 (7.7) | 1,465 (19.5) |
| Asian, non-Hispanic | 7 (10.9) | 4 (7.0) | 2 (4.1) | 0 (0.0) | 6 (15.4) | 700 (9.3) |
| Other or multiple races | 1 (1.6) | 3 (5.3) | 3 (6.1) | 2 (4.8) | 0 (0.0) | 273 (3.6) |
| Unknown race, non-      Hispanic | 0 (0.0) | 2 (3.5) | 0 (0.0) | 1 (2.4) | 0 (0.0) | 153 (2.0) |
| **Residential address Social Vulnerability Index (SVI), no. (%)**^**f**^ |  |  |  |  |  |  |
| High social vulnerability^g^ | 3 (4.8) | 17 (31.5) | 12 (26.7) | 5 (12.8) | 4 (13.3) | 1,681 (24.2) |
| Low social vulnerability^h^ | 32 (50.8) | 14 (25.9) | 8 (17.8) | 18 (46.2) | 14 (46.7) | 1,804 (26.0) |
| **Community exposures, no. (%)** |  |  |  |  |  |  |
| Traveled domestically or      internationally | 22 (34.4) | 7 (12.3) | 9 (18.4) | 5 (11.9) | 14 (35.9) | 1,157 (15.4) |
| Attended a mass gathering or      gathering with people other than        household members | 26 (40.6) | 6 (10.6) | 14 (28.6) | 8 (19.1) | 14 (35.9) | 1,818 (24.1) |
| Used public or shared      transportation | 13 (20.3) | 6 (10.5) | 9 (18.4) | 2 (4.8) | 14 (35.9) | 1,058 (14.1) |
| Had close contact with ill person(s)        outside of a healthcare facility | 13 (20.3) | 9 (15.8) | 10 (20.4) | 10 (23.8) | 10 (25.6) | 1,412 (18.8) |
| Had close contact with a family      member(s) who had COVID-19 | 16 (25.0) | 12 (21.1) | 13 (26.5) | 12 (28.6) | 18 (46.2) | 1,883 (25.0) |
| **Underlying conditions, no. (%)** |  |  |  |  |  |  |
| At least one underlying condition | 30 (46.9) | 41 (71.9) | 33 (67.4) | 27 (64.3) | 19 (48.7) | 4,660 (61.9) |
| Asthma | 13 (20.3) | 11 (19.3) | 3 (6.1) | 6 (14.3) | 4 (10.3) | 1,051 (14.0) |
| Autoimmune or      rheumatologic disease | 5 (7.8) | 1 (1.8) | 1 (2.0) | 2 (4.8) | 1 (2.6) | 351 (4.7) |
| Chronic kidney disease | 0 (0.0) | 0 (0.0) | 0 (0.0) | 0 (0.0) | 0 (0.0) | 31 (0.4) |
| Chronic obstructive      pulmonary disease | 0 (0.0) | 0 (0.0) | 0 (0.0) | 0 (0.0) | 0 (0.0) | 46 (0.6) |
| Current or recent smoker^i^ | 4 (6.3) | 14 (24.6) | 13 (26.5) | 5 (11.9) | 7 (18.0) | 1,628 (21.6) |
| Diabetes mellitus | 0 (0.0) | 6 (10.5) | 4 (8.2) | 1 (2.4) | 0 (0.0) | 471 (6.3) |
| Heart condition | 3 (4.7) | 2 (3.5) | 2 (4.1) | 1 (2.4) | 5 (12.8) | 249 (3.3) |
| Hypertension | 1 (1.6) | 10 (17.5) | 5 (10.2) | 4 (9.5) | 2 (5.1) | 1,163 (15.4) |
| Obesity or severe obesity | 7 (10.9) | 30 (52.6) | 18 (36.7) | 19 (45.2) | 11 (28.2) | 2,549 (33.9) |
| Pregnancy | 5 (7.8) | 0 (0.0) | 0 (0.0) | 0 (0.0) | 1 (2.6) | 160 (2.1) |
| **COVID-19 vaccination status at time of test, no. (%)**^**j**^ |  |  |  |  |  |  |
| Vaccinated | 20 (87.0) | 14 (70.0) | 10 (71.4) | 8 (100) | 10 (100) | 1,541 (73.6) |
| Unvaccinated | 3 (13.0) | 6 (30.0) | 4 (28.6) | 0 (0.0) | 0 (0.0) | 548 (26.2) |

Table S1. Characteristics of healthcare personnel with SARS-CoV-2 infection, by primary healthcare role, 2020–2021 (continued)

|  | **Student** **(n=36)** | **Transport personnel (n=36)** | **Dietician**^**v**^ **(n=34)** | **Other licensed healthcare professional (n=31)** | **Coordinator or case manager (n=29)** | **All professions** **(N=7,531)** |
| --- | --- | --- | --- | --- | --- | --- |
| **Facility type, no. (%)** |  |  |  |  |  |  |
| Hospital | 24 (66.7) | 26 (72.2) | 21 (61.8) | 10 (32.3) | 11 (37.9) | 3,975 (52.8) |
| Nursing home | 2 (5.6) | 1 (2.8) | 8 (23.5) | 1 (3.2) | 2 (6.9) | 1,142 (15.2) |
| Outpatient clinic | 6 (16.7) | 3 (8.3) | 3 (8.8) | 18 (58.1) | 5 (17.2) | 1,223 (16.2) |
| Home healthcare setting | 0 (0.0) | 0 (0.0) | 0 (0.0) | 0 (0.0) | 3 (10.3) | 386 (5.1) |
| Assisted living facility | 0 (0.0) | 1 (2.8) | 0 (0.0) | 0 (0.0) | 3 (10.3) | 126 (1.7) |
| Other facilities^b^ | 4 (11.1) | 5 (13.9) | 2 (5.9) | 2 (6.5) | 5 (17.2) | 679 (9.0) |
| **Sex, no, (%)**^**c**^ |  |  |  |  |  |  |
| Female | 31 (86.1) | 17 (47.2) | 34 (100) | 22 (71.0) | 25 (86.2) | 5,933 (78.8) |
| Male | 5 (13.9) | 19 (52.8) | 0 (0.0) | 9 (29.0) | 4 (13.8) | 1,574 (20.9) |
| **Age group in years, no. (%)**^**d**^ |  |  |  |  |  |  |
| Median [IQR] | 25 [24–29] | 41 [26–55] | 38 [28–49] | 39 [31–54] | 42 [35–50] | 39 [30–50] |
| <30 | 27 (75.0) | 14 (38.9) | 12 (35.3) | 5 (16.1) | 2 (6.9) | 1,769 (23.5) |
| ≥30 | 9 (25.0) | 22 (61.1) | 22 (64.7) | 26 (83.9) | 27 (93.1) | 5,686 (75.5) |
| **Race and ethnicity, no. (%)**^**e**^ |  |  |  |  |  |  |
| Hispanic | 5 (13.9) | 6 (16.7) | 5 (14.7) | 4 (12.9) | 11 (37.9) | 1,495 (19.9) |
| White, non-Hispanic | 20 (55.6) | 9 (25.0) | 22 (64.7) | 21 (67.7) | 10 (34.5) | 3,445 (45.7) |
| Black or African American, non-Hispanic | 5 (13.9) | 15 (41.7) | 3 (8.8) | 2 (6.5) | 5 (17.2) | 1,465 (19.5) |
| Asian, non-Hispanic | 4 (11.1) | 3 (8.3) | 2 (5.9) | 3 (9.7) | 2 (6.9) | 700 (9.3) |
| Other or multiple races | 0 (0.0) | 0 (0.0) | 0 (0.0) | 1 (3.2) | 1 (3.5) | 273 (3.6) |
| Unknown race, non-Hispanic | 2 (5.6) | 3 (8.3) | 2 (5.9) | 0 (0.0) | 0 (0.0) | 153 (2.0) |
| **Residential address Social Vulnerability Index (SVI), no. (%)**^**f**^ |  |  |  |  |  |  |
| High social vulnerability^g^ | 3 (8.6) | 12 (37.5) | 6 (18.8) | 2 (7.1) | 8 (28.6) | 1,681 (24.2) |
| Low social vulnerability^h^ | 13 (37.1) | 7 (21.9) | 10 (31.3) | 8 (28.6) | 6 (21.4) | 1,804 (26.0) |
| **Community exposures, no. (%)** |  |  |  |  |  |  |
| Traveled domestically or      internationally | 6 (16.7) | 0 (0.0) | 5 (14.7) | 6 (19.4) | 5 (17.2) | 1,157 (15.4) |
| Attended a mass gathering or gathering      with people other than household      members | 10 (27.8) | 5 (13.9) | 13 (38.2) | 8 (25.8) | 7 (24.1) | 1,818 (24.1) |
| Used public or shared transportation | 4 (11.1) | 3 (8.3) | 5 (14.7) | 3 (9.7) | 5 (17.2) | 1,058 (14.1) |
| Had close contact with ill person(s) outside      of a healthcare facility | 4 (11.1) | 4 (11.1) | 7 (20.6) | 5 (16.1) | 7 (24.1) | 1,412 (18.8) |
| Had close contact with a family member(s)      who had COVID-19 | 8 (22.2) | 8 (22.2) | 12 (35.3) | 7 (22.6) | 12 (41.4) | 1,883 (25.0) |
| **Underlying conditions, no. (%)** |  |  |  |  |  |  |
| At least one underlying condition | 10 (27.8) | 21 (58.3) | 19 (55.9) | 15 (48.4) | 22 (75.9) | 4,660 (61.9) |
| Asthma | 3 (8.3) | 2 (5.6) | 7 (20.6) | 4 (12.9) | 5 (17.2) | 1,051 (14.0) |
| Autoimmune or rheumatologic disease | 0 (0.0) | 1 (2.8) | 4 (11.8) | 0 (0.0) | 2 (6.9) | 351 (4.7) |
| Chronic kidney disease | 0 (0.0) | 0 (0.0) | 0 (0.0) | 0 (0.0) | 0 (0.0) | 31 (0.4) |
| Chronic obstructive pulmonary disease | 0 (0.0) | 1 (2.8) | 0 (0.0) | 0 (0.0) | 1 (3.5) | 46 (0.6) |
| Current or recent smoker^i^ | 1 (2.8) | 5 (13.9) | 5 (14.7) | 6 (19.4) | 9 (31.0) | 1,628 (21.6) |
| Diabetes mellitus | 0 (0.0) | 5 (13.9) | 4 (11.8) | 0 (0.0) | 3 (10.3) | 471 (6.3) |
| Heart condition | 1 (2.8) | 2 (5.6) | 1 (2.9) | 0 (0.0) | 2 (6.9) | 249 (3.3) |
| Hypertension | 1 (2.8) | 7 (19.4) | 4 (11.8) | 2 (6.5) | 5 (17.2) | 1,163 (15.4) |
| Obesity or severe obesity | 6 (16.7) | 17 (47.2) | 5 (14.7) | 6 (19.4) | 13 (44.8) | 2,549 (33.9) |
| Pregnancy | 0 (0.0) | 0 (0.0) | 3 (8.8) | 1 (3.2) | 0 (0.0) | 160 (2.1) |
| **COVID-19 vaccination status at time** **of test, no. (%)**^**j**^ |  |  |  |  |  |  |
| Vaccinated | 5 (62.5) | 4 (44.4) | 7 (87.5) | 6 (42.9) | 6 (66.7) | 1,541 (73.6) |
| Unvaccinated | 3 (37.5) | 5 (55.6) | 1 (12.5) | 8 (57.1) | 3 (33.3) | 548 (26.2) |

Table S1. Characteristics of healthcare personnel with SARS-CoV-2 infection, by primary healthcare role, 2020–2021 (continue) 

|  | **Speech therapist** **(n=27)** | **Patient screener or greeter** **(n=22)** | **Activities coordinator** **(n=15)** | **Chaplain** **(n=10)** | **Other professions or preferred not to answer** **(n=138)** | **All professions** **(N=7,531)** |
| --- | --- | --- | --- | --- | --- | --- |
| **Facility type, no. (%)** |  |  |  |  |  |  |
| Hospital | 12 (44.4) | 15 (68.2) | 0 (0.0) | 7 (70.0) | 65 (47.1) | 3,975 (52.8) |
| Nursing home | 8 (29.6) | 0 (0.0) | 12 (80.0) | 1 (10.0) | 14 (10.1) | 1,142 (15.2) |
| Outpatient clinic | 6 (22.2) | 5 (22.7) | 0 (0.0) | 0 (0.0) | 24 (17.4) | 1,223 (16.2) |
| Home healthcare setting | 0 (0.0) | 0 (0.0) | 0 (0.0) | 0 (0.0) | 5 (3.6) | 386 (5.1) |
| Assisted living facility | 0 (0.0) | 0 (0.0) | 1 (6.7) | 0 (0.0) | 0 (0.0) | 126 (1.7) |
| Other facilities^b^ | 1 (3.7) | 2 (9.1) | 2 (13.3) | 2 (20.0) | 30 (21.7) | 679 (9.0) |
| **Sex, no, (%)**^**c**^ |  |  |  |  |  |  |
| Female | 25 (92.6) | 14 (63.6) | 12 (80.0) | 5 (50.0) | 96 (69.6) | 5,933 (78.8) |
| Male | 2 (7.4) | 8 (36.4) | 3 (20.0) | 5 (50.0) | 40 (29.0) | 1,574 (20.9) |
| **Age group in years, no. (%)**^**d**^ |  |  |  |  |  |  |
| Median [IQR] | 37 [32–48] | 30 [22–44] | 33 [28–58] | 58 [38–69] | 36 [29–50] | 39 [30–50] |
| <30 | 5 (18.5) | 11 (50.0) | 6 (40.0) | 1 (10.0) | 35 (25.4) | 1,769 (23.5) |
| ≥30 | 22 (81.5) | 11 (50.0) | 9 (60.0) | 9 (90.0) | 95 (68.8) | 5,686 (75.5) |
| **Race and ethnicity, no. (%)**^**e**^ |  |  |  |  |  |  |
| Hispanic | 3 (11.1) | 8 (36.4) | 5 (33.3) | 0 (0.0) | 33 (23.9) | 1,495 (19.9) |
| White, non-Hispanic | 18 (66.7) | 8 (36.4) | 4 (26.7) | 9 (90.0) | 51 (37.0) | 3,445 (45.7) |
| Black or African American, non-Hispanic | 3 (11.1) | 3 (13.6) | 2 (13.3) | 1 (10.0) | 28 (20.3) | 1,465 (19.5) |
| Asian, non-Hispanic | 3 (11.1) | 2 (9.1) | 3 (20.0) | 0 (0.0) | 13 (9.4) | 700 (9.3) |
| Other or multiple races | 0 (0.0) | 1 (4.6) | 1 (6.7) | 0 (0.0) | 4 (2.9) | 273 (3.6) |
| Unknown race, non-Hispanic | 0 (0.0) | 0 (0.0) | 0 (0.0) | 0 (0.0) | 9 (6.5) | 153 (2.0) |
| **Residential address Social Vulnerability Index (SVI), no. (%)**^**f**^ |  |  |  |  |  |  |
| High social vulnerability^g^ | 2 (8.3) | 7 (36.8) | 7 (50.0) | 2 (22.2) | 29 (22.3) | 1,681 (24.2) |
| Low social vulnerability^h^ | 11 (45.8) | 3 (15.8) | 1 (7.1) | 3 (33.3) | 41 (31.5) | 1,804 (26.0) |
| **Community exposures, no. (%)** |  |  |  |  |  |  |
| Traveled domestically or internationally | 7 (25.9) | 5 (22.7) | 3 (20.0) | 4 (40.0) | 26 (18.8) | 1,157 (15.4) |
| Attended a mass gathering or gathering      with people other than household      members | 8 (29.6) | 2 (9.1) | 5 (33.3) | 4 (40.0) | 40 (29.2) | 1,818 (24.1) |
| Used public or shared transportation | 7 (25.9) | 4 (18.2) | 2 (13.3) | 4 (40.0) | 29 (21.0) | 1,058 (14.1) |
| Had close contact with ill person(s) outside      of a healthcare facility | 8 (29.6) | 5 (22.7) | 2 (13.3) | 1 (10.0) | 24 (17.5) | 1,412 (18.8) |
| Had close contact with a family member(s)      who had COVID-19 | 6 (22.2) | 5 (22.7) | 1 (6.7) | 1 (10.0) | 31 (22.5) | 1,883 (25.0) |
| **Underlying conditions, no. (%)** |  |  |  |  |  |  |
| At least one underlying condition | 13 (48.2) | 10 (45.5) | 14 (93.3) | 7 (70.0) | 82 (59.4) | 4,660 (61.9) |
| Asthma | 3 (11.1) | 0 (0.0) | 4 (26.7) | 2 (20.0) | 26 (18.8) | 1,051 (14.0) |
| Autoimmune or rheumatologic disease | 2 (7.4) | 1 (4.6) | 0 (0.0) | 0 (0.0) | 4 (2.9) | 351 (4.7) |
| Chronic kidney disease | 0 (0.0) | 0 (0.0) | 0 (0.0) | 0 (0.0) | 0 (0.0) | 31 (0.4) |
| Chronic obstructive pulmonary disease | 0 (0.0) | 0 (0.0) | 0 (0.0) | 0 (0.0) | 0(0.0) | 46 (0.6) |
| Current or recent smoker^i^ | 2 (7.4) | 5 (22.7) | 7 (46.7) | 3 (30.0) | 34 (24.6) | 1,628 (21.6) |
| Diabetes mellitus | 0 (0.0) | 1 (4.6) | 2 (13.3) | 0 (0.0) | 10 (7.3) | 471 (6.3) |
| Heart condition | 1 (3.7) | 0 (0.0) | 0 (0.0) | 2 (20.0) | 3 (2.2) | 249 (3.3) |
| Hypertension | 4 (14.8) | 1 (4.6) | 4 (26.7) | 3 (30.0) | 19 (13.8) | 1,163 (15.4) |
| Obesity or severe obesity | 6 (22.2) | 6 (27.3) | 9 (60.0) | 1 (10.0) | 42 (30.4) | 2,549 (33.9) |
| Pregnancy | 0 (0.0) | 0 (0.0) | 0 (0.0) | 0 (0.0) | 0 (0.0) | 160 (2.1) |
| **COVID-19 vaccination status at time of test, no. (%)**^**j**^ |  |  |  |  |  |  |
| Vaccinated | 6 (85.7) | 6 (75.0) | 5 (83.3) | 4 (80.0) | 36 (67.9) | 1,541 (73.6) |
| Unvaccinated | 1 (14.3) | 2 (25.0) | 1 (16.7) | 1 (20.0) | 16 (30.2) | 548 (26.2) |

^a^HCP who reported their role as administrative personnel, director, financial personnel, human resources personnel, receptionist, patient service assistant, clinical supervisor, or marketing personnel.

^b^Other facilities include: administrative building, correctional facility, dental facility, outpatient dialysis unit, emergency medical service, free standing emergency room, hospice facility, laboratory, memory care facility, mental health facility, pharmacy, public health department, rehabilitation center, school, COVID-19 testing center, urgent care center.

^c^24 HCP did not answer question about sex or reported sex as unknown.

^d^76 HCP did not answer question about age.

^e^168 HCP with missing or unknown ethnicity were grouped as non-Hispanic.

^f^Census tract-level SVI for HCP residential address. 592 HCP were not matched with SVI data due to lack of valid addresses or residential addresses that were out of catchment areas.  

^g^Highest quartile of SVI values for census tracts where healthcare personnel resided.

^h^Lowest quartile of SVI values for census tracts where healthcare personnel resided.

^i^Recent smokers were defined as HCP who quit smoking <1 year before the interview date.

^j^Among 2,094 HCP cases who tested positive for SARS-CoV-2 in 2021; 5 HCP reported unknown COVID-19 vaccination status; vaccinated is defined as having received at least one dose of COVID-19 vaccine ≥14 days before the SARS-CoV-2 positive test dates.

^k^HCP who reported their role as facilities personnel, electrician, or engineer.

^l^HCP who reported their role as environmental services, laundry, or housekeeping personnel.

^m^HCP who reported their role as surgical or medical technician, ambulatory technician, dialysis technician, or radiologist therapist.

^n^HCP who reported their role as food services personnel, chef, or dietary aide.

^o^HCP who reported their role as pharmacist or pharmacist interns.

^p^HCP who reported their role as laboratory personnel, histotechnologist, or laboratory supervisor.  

^q^HCP who reported their role as radiology personnel, cardiac sonographer, CT technician, MRI technician,

mammographer, or X-ray technician.

^r^HCP who reported their role as emergency medical services personnel, paramedic personnel, fire fighter, national guard, or emergency medical technician. 

^s^HCP who reported their role as mental health personnel, behavioral technician, addictions counselor, mental health technician, or clinical psychologist. 

^t^HCP who reported their role as dental practitioner, dental assistant, dental hygienist, or hygienist assistant.

^u^HCP who reported their role as equipment technician, biomedical equipment technician, cardiac monitor technician, supply technician, telemetry technician, or sterile processing technician.

^v^HCP who reported their role as dietitian or nutrition assistant. 

Table S2. Percentage of healthcare personnel with SARS-CoV-2 infection living in high or low Social Vulnerability Index census tracts, by Emerging Infections Program site and primary healthcare role, 2020–2021

| **Site, no. (%)** |  | **All Professions**^**a**^ | **Registered Nurse** | **Administrative Personnel**^**b**^ | **Certified Nursing Assistant** | **Physician** | **Medical Assistant** | **Home Healthcare worker** | **Other Professions**^**c**^ |
| --- | --- | --- | --- | --- | --- | --- | --- | --- | --- |
| EIP^d ^1 | High social vulnerability^e^ | 50 (16.2) | 10 (9.9) | 6 (18.2) | 6 (30.0) | 2 (6.7) | 3 (18.8) | 1 (20.0) | 22 (21.2) |
|  | Low social vulnerability^f^ | 113 (36.6) | 45 (44.6) | 9 (27.3) | 6 (30.0) | 17 (56.7) | 4 (25.0) | 1 (20.0) | 31 (29.8) |
| EIP 2 | High social vulnerability | 60 (21.0) | 15 (15.6) | 7 (14.9) | 5 (41.7) | 0 (0.0) | 7 (50.0) | 0 (0.0) | 26 (24.1) |
|  | Low social vulnerability | 49 (17.1) | 22 (22.9) | 7 (14.9) | 1 (8.3) | 4 (50.0) | 2 (14.3) | 0 (0.0) | 13 (12.0) |
| EIP 3 | High social vulnerability | 59 (18.0) | 15 (13.2) | 7 (21.9) | 4 (44.4) | 5 (12.8) | 6 (40.0) | 1 (100) | 21 (17.8) |
|  | Low social vulnerability | 130 (39.6) | 53 (46.5) | 8 (25.0) | 4 (44.4) | 14 (35.9) | 4 (26.7) | 0 (0.0) | 47 (39.8) |
| EIP 4 | High social vulnerability | 231 (16.9) | 38 (13.1) | 40 (18.9) | 33 (27.0) | 2 (3.6) | 21 (25.0) | 8 (25.0) | 89 (15.6) |
|  | Low social vulnerability | 472 (34.6) | 123 (42.6) | 57 (26.9) | 23 (18.9) | 35 (62.5) | 18 (21.4) | 8 (25.0) | 208 (36.5) |
| EIP 5 | High social vulnerability | 130 (23.2) | 23 (13.5) | 15 (24.2) | 8 (21.6) | 2 (9.5) | 28 (50.9) | -- | 54 (25.1) |
|  | Low social vulnerability | 163 (29.1) | 62 (36.5) | 10 (16.1) | 7 (18.9) | 11 (52.4) | 11 (20.0) | -- | 62 (28.8) |
| EIP 6 | High social vulnerability | 26 (12.1) | 3 (4.2) | 6 (17.6) | 5 (33.3) | 3 (25.0) | 0 (0.0) | -- | 9 (11.5) |
|  | Low social vulnerability | 97 (45.1) | 40 (56.3) | 13 (38.2) | 7 (46.7) | 6 (50.0) | 2 (40.0) | -- | 29 (37.2) |
| EIP 7 | High social vulnerability | 105 (16.0) | 26 (14.4) | 12 (13.8) | 12 (27.9) | 2 (3.3) | 2 (25.0) | 0 (0.0) | 51 (18.5) |
|  | Low social vulnerability | 247 (37.6) | 72 (39.8) | 24 (27.6) | 6 (14.0) | 42 (68.9) | 1 (12.5) | 0 (0.0) | 102 (37.0) |
| EIP 8 | High social vulnerability | 240 (27.1) | 13 (6.9) | 20 (25.3) | 116 (58.3) | 3 (7.0) | 3 (23.1) | 1 (100) | 84 (23.3) |
|  | Low social vulnerability | 216 (24.4) | 63 (33.3) | 20 (25.3) | 21 (10.6) | 19 (44.2) | 3 (23.1) | 0 (0.0) | 90 (24.9) |
| EIP 9 | High social vulnerability | 689 (35.1) | 62 (22.3) | 73 (32.7) | 81 (41.3) | 7 (9.5) | 67 (45.9) | 155 (47.5) | 244 (34.0) |
|  | Low social vulnerability | 263 (13.4) | 51 (18.3) | 23 (10.3) | 13 (6.6) | 37 (50.0) | 8 (5.5) | 26 (8.0) | 105 (14.6) |
| EIP 10 | High social vulnerability | 91 (24.4) | 20 (13.9) | 8 (21.6) | 13 (50.0) | 3 (13.0) | 1 (12.5) | -- | 46 (34.1) |
|  | Low social vulnerability | 54 (14.5) | 25 (17.4) | 4 (10.8) | 1 (3.8) | 8 (34.8) | 2 (25.0) | -- | 14 (10.4) |
| All EIP sites | High social vulnerability | 1,681 (24.2) | 225 (13.8) | 194 (22.9) | 283 (41.7) | 29 (7.9) | 138 (37.9) | 166 (45.2) | 646 (24.1) |
|  | Low social vulnerability | 1,804 (26.0) | 556 (34.1) | 175 (20.7) | 89 (13.1) | 193 (52.6) | 55 (15.1) | 35 (9.5) | 701 (26.1) |

^a^592 HCP were not matched with SVI data due to lack of valid addresses or residential addresses that were out of catchment areas.  

^b^HCP who reported their role as administrative personnel, director, financial personnel, human resources personnel, receptionist, patient service assistant, clinical supervisor, or marketing personnel.

^c^All other professions include: roles such as licensed practical nurse, nurse practitioner, facilities personnel, environmental services personnel, food services personnel, surgical or medical technician, laboratory personnel, pharmacist, or radiology personnel. 

^d^Emerging Infections Program. 

^e^Highest quartile of SVI values for census tracts where healthcare personnel resided.

^f^Lowest quartile of SVI values for census tracts where healthcare personnel resided.

Table S3. Personal protective equipment use, workplace exposures, and patient care activities among healthcare personnel with SARS-CoV-2 infection and close contact with patients with COVID-19 in healthcare settings, by primary healthcare role, 2020–2021

|  | **Licensed Practical Nurse** **(n=121)** | **Nurse Practitioner** **(n=73)** |  | **Surgical or Medical Technicians**^**b**^ **(n=72)** | **Environmental Services Personnel**^**c**^ **(n=28)** | **Facilities Personnel**^**d**^ **(n=27)** | **Food Services Personnel**^**e**^ **(n=7)** | **All professions** **(n=2,606)** |  |
| --- | --- | --- | --- | --- | --- | --- | --- | --- | --- |
| **Facility type, no. (%)** |  |  |  |  |  |  |  |  |  |
| Hospital | 19 (15.7) | 37 (50.7) |  | 60 (83.3) | 16 (57.1) | 23 (85.2) | 3 (42.9) | 1,631 (62.6) |  |
| Nursing home | 73 (60.3) | 8 (11.0) |  | 2 (2.8) | 11 (39.3) | 2 (7.4) | 2 (28.6) | 504 (19.3) |  |
| Outpatient clinic | 7 (5.8) | 15 (20.6) |  | 3 (4.2) | 1 (3.6) | 2 (7.4) | 0 (0.0) | 156 (6.0) |  |
| Home healthcare setting | 2 (1.7) | 1 (1.4) |  | 0 (0.0) | 0 (0.0) | 0 (0.0) | 0 (0.0) | 86 (3.3) |  |
| Assisted living facility | 4 (3.3) | 0 (0.0) |  | 4 (5.6) | 0 (0.0) | 0 (0.0) | 2 (28.6) | 42 (1.6) |  |
| Other facilities | 16 (13.2) | 12 (16.4) |  | 3 (4.2) | 0 (0.0) | 0 (0.0) | 0 (0.0) | 187 (7.2) |  |
| **PPE use and exposures in healthcare setting, no. (%)** |  |  |  |  |  |  |  |  |  |
| Used gloves all the time | 107 (88.4) | 63 (86.3) |  | 67 (93.1) | 26 (92.9) | 22 (81.5) | 5 (71.4) | 2,224 (85.5) |  |
| Used a mask or      respirator all the time | 109 (90.1) | 63 (86.3) |  | 68 (94.4) | 27 (96.4) | 26 (96.3) | 0 (0.0) | 2,392 (91.8) |  |
| Used goggles or a face       shield all the time | 82 (67.8) | 56 (76.7) |  | 57 (79.2) | 17 (60.7) | 17 (63.0) | 3 (42.9) | 1,759 (67.7) |  |
| Used a gown all the time | 79 (65.3) | 52 (71.2) |  | 50 (70.4) | 19 (67.9) | 14 (51.9) | 5 (71.4) | 1,625 (62.6) |  |
| Always cared for COVID-      19 patients who had      source control in place | 16 (13.2) | 27 (37.0) |  | 31 (43.1) | 2 (7.1) | 8 (29.6) | 1 (14.3) | 527 (20.2) |  |
| Had a mucous membrane      or skin exposure to body      fluids from a COVID-19      patient | 21 (17.4) | 12 (16.9) |  | 9 (12.5) | 1 (3.6) | 1 (3.7) | 1 (14.3) | 495 (19.1) |  |
| Practiced extended use or      reuse of a respirator | 58 (47.9) | 49 (67.1) |  | 46 (63.9) | 12 (42.9) | 7 (25.9) | 0 (0.0) | 1,507 (57.8) |  |
| Always followed hand        hygiene recommendations      during care of COVID-19      patients | 108 (89.3) | 65 (91.6) |  | 66 (91.7) | 25 (89.3) | 23 (85.2) | 6 (85.7) | 2,346 (90.4) |  |
| **Patient care activities, no. (%)** |  |  |  |  |  |  |  |  |  |
| Assisted patients with       COVID-19 with activities      of daily living | 85 (70.3) | 18 (24.7) |  | 42 (58.3) | 4 (14.3) | 8 (29.6) | 3 (42.9) | 1,716 (65.9) |  |
| Bathing^a^ | 41 (48.2) | 5 (27.8) |  | 12 (28.6) | 0 (0.0) | 0 (0.0) | 0 (0.0) | 863 (50.3) |  |
| Emptying bedpan^a^ | 34 (40.0) | 2 (11.1) |  | 14 (33.3) | 3 (75.0) | 2 (25.0) | 0 (0.0) | 766 (44.6) |  |
| Feeding^a^ | 49 (57.7) | 6 (33.3) |  | 10 (23.8) | 1 (25.0) | 0 (0.0) | 0 (0.0) | 807 (47.0) |  |
| Lifting or positioning^a^ | 79 (92.9) | 0 (0.0) |  | 39 (92.9) | 0 (0.0) | 2 (25.0) | 0 (0.0) | 1,564 (91.1) |  |
| Performing oral care^a^ | 18 (21.2) | 2 (11.1) |  | 5 (11.9) | 0 (0.0) | 0 (0.0) | 0 (0.0) | 392 (22.8) |  |
| Other activities of daily           living^a^ | 12 (14.1) | 3 (16.7) |  | 17 (40.5) | 0 (0.0) | 5 (62.5) | 1 (33.3) | 410 (23.9) |  |
| Provided non-procedure      clinical care to patients      with COVID-19 | 111 (91.7) | 64 (87.7) |  | 42 (58.3) | 2 (7.1) | 4 (14.8) | 0 (0.0) | 1,963 (75.3) |  |
| Performed procedures        on patients with COVID-19 | 76 (62.8) | 19 (26.0) |  | 44 (61.1) | 0 (0.0) | 0 (0.0) | 0 (0.0) | 1,199 (46.0) |  |
| Performed environmental      cleaning activities in COVID-19      patient care area | 64 (52.9) | 8 (11.0) |  | 28 (38.9) | 26 (92.9) | 12 (44.4) | 0 (0.0) | 1,277 (49.0) |  |
| Provided respiratory care to      patients with COVID-19 | 72 (59.5) | 34 (46.6) |  | 26 (36.1) | 0 (0.0) | 0 (0.0) | 0 (0.0) | 1,211 (46.5) |  |
| Performed administrative       activities with patients with      COVID-19 | 3 (2.5) | 4 (5.5) |  | 2 (2.8) | 0 (0.0) | 8 (29.6) | 5 (71.4) | 217 (8.3) |  |

^a^Among HCP who assisted patients with COVID-19 with activities of daily living.

^b^HCP who reported their role as surgical or medical technician, ambulatory technician, dialysis technician, or radiologist therapist.

^c^HCP who reported their role as environmental services, laundry, or housekeeping personnel.

^d^HCP who reported their role as facilities personnel, electrician, or engineer.

^e^HCP who reported their role as food services personnel, chef, or dietary aide.
